# From adaptive resolution to molecular dynamics of open systems

**DOI:** 10.1140/epjb/s10051-021-00193-w

**Published:** 2021-09-23

**Authors:** Robinson Cortes-Huerto, Matej Praprotnik, Kurt Kremer, Luigi Delle Site

**Affiliations:** 1 Max Planck Institute for Polymer Research, Ackermannweg 10, 55128 Mainz, Germany; 2 Laboratory for Molecular Modeling, National Institute of Chemistry, Ljubljana, Slovenia and Department of Physics, Faculty of Mathematics and Physics, University of Ljubljana, Ljubljana, Slovenia; 3 Department of Mathematics and Computer Science, Institute for Mathematics, Freie Universität Berlin, Berlin, Germany

## Abstract

**Abstract:**

We provide an overview of the Adaptive Resolution Simulation method (AdResS) based on discussing its basic principles and presenting its current numerical and theoretical developments. Examples of applications to systems of interest to soft matter, chemical physics, and condensed matter illustrate the method’s advantages and limitations in its practical use and thus settle the challenge for further future numerical and theoretical developments.

**Graphic abstract:**

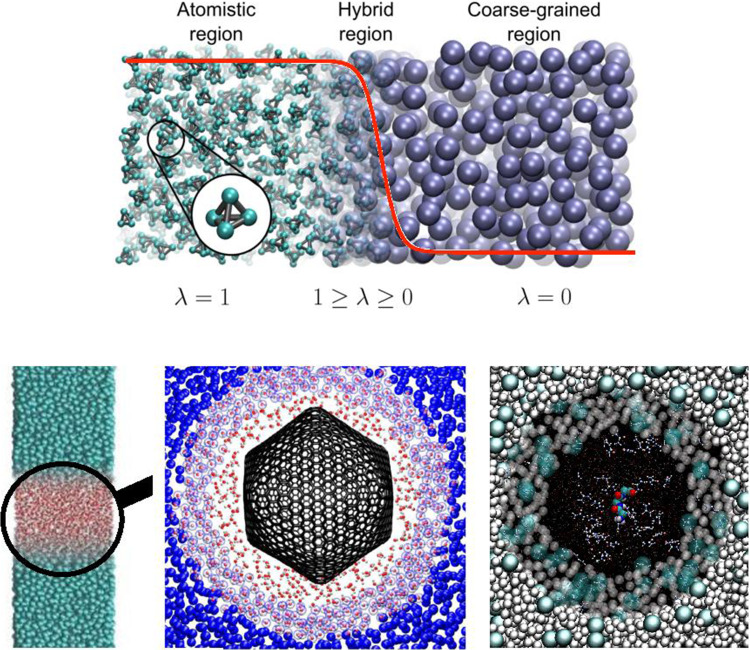

## Introduction

Computer simulations of physical model systems, materials or simple molecules date back to the 50s of the last century. Early pioneers started with the very first molecular dynamics or Monte Carlo simulations of simple hard-sphere liquids or lattice models for polymers (for a historical account, see Ref. [1]). While these first in silico experiments at the time were genuinely exploratory, their applicability, of course, was strongly hampered by the limitations of available soft- and hard-ware. Since then, dramatic improvements in hardware and simulation methodology allowed researchers to systematically study far more complex systems on space and time scales closer to realistic systems and processes. Because of this, modern computer simulations play a central role in many disciplines nowadays. This development continues with new numerical approaches, including modern machine learning techniques and the advent of exascale computing on the horizon. It has to be seen whether there will be another boost by quantum computing in the not far future [2].

**Fig. 1:**
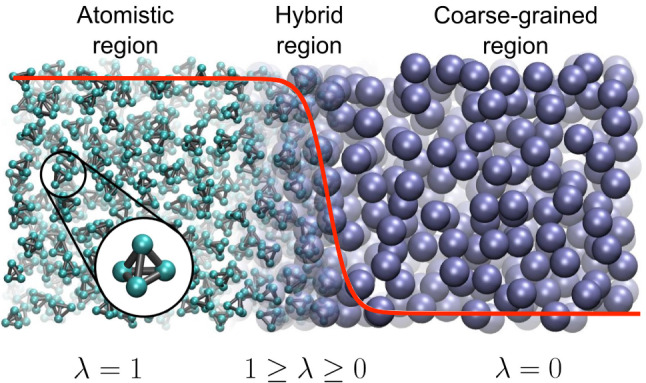
Original AdResS [5, 6] setup applied to a test system of a liquid of tetrahedral molecules coupled to a single particle representation of these molecules. As indicated the system consists of an atomistic region ($\lambda = 1)$, a coarse grained region ($\lambda = 0$) and the interpolating hybrid region ($0< \lambda < 1$). Reprinted figure with permission from Ref. [9]. Copyright (2013) by the American Physical Society

Still, despite all that huge progress, we are facing many real challenges. There is the severe problem of scales, which can be found in all fields of (materials) modeling. In (too) simple terms, one can take two opposing views. For a physicist, a generic, typically reduced picture provides understanding and reveals mechanisms, while for a chemist, all the local details count and result in a material or a function. Of course, both are true, and different views apply depending on the question under consideration. However, problems at the interface between these two ways of thinking pose the most interesting scientific questions. Namely, conditions where local details are decisive, that couple to large scale environments where most details average out into a more simple generic model. These conditions hold for many complex systems but especially for biological and synthetic soft matter. It is often the case that relatively small local changes, e.g., molecular structure, can have significant consequences for global system properties or function. To deal with that numerically, typically simulations of models of different levels of resolution are performed independently, and the results are compared and linked to each other [3]. Especially when dealing with long time dynamical problems, e.g. polymer melt dynamics, this has been a successful strategy. In such cases all atom dynamics has been successfully used to calibrate time scales of simple bead spring chain models. However, already for liquid crystalline systems, this direct approach does not work anymore [3, 4]. In other cases, one would like to study local properties, which need the contact to a larger reservoir for which one not necessarily has to take all the details permanently into account. Thus it is advantageous to have simulation methods at hand, which deal locally with chemically specific problems, where the microscopic central part is in equilibrium with a simplified, coarse-grained (cg or CG) environment. Moreover, particles can freely move without experiencing a free energy barrier between these regions where different descriptions are considered. The adaptive resolution simulation (AdResS) approach provides such a framework [5–10]. The original test setup for the first simulation is illustrated in Fig. 1.

**Fig. 2:**
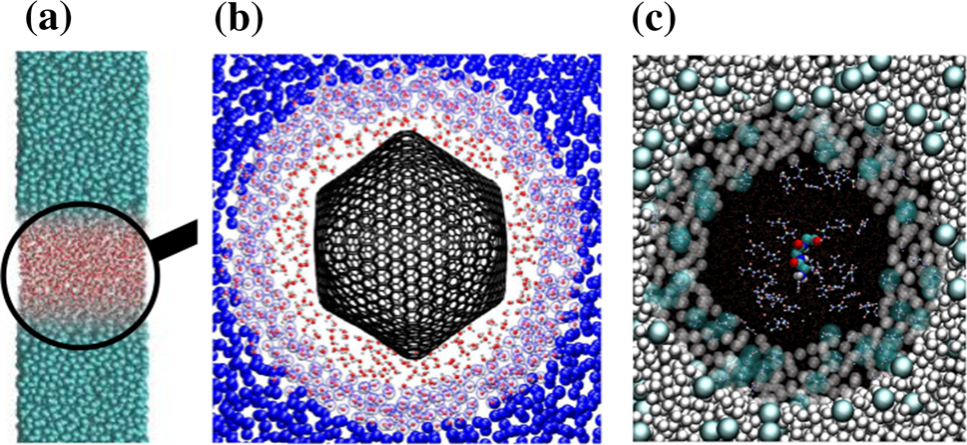
Typical scenarios, where AdResS type of simulations can be useful. In **a** coarse grained simulation of water is run and the AdResS setup is used to zoom into the liquid like with a magnification glass allowing for a more detailed analysis. In **b** the solvation shell of a huge fullerene in water is studied. The variation of the thickness of the atomistic water layer allows to shed light onto the structuring of that layer by the fullerene on one side and the bulk water on the other side. Reprinted from Ref. [12], with the permission of AIP Publishing. **c** Is from a study of tri-glycine in a water urea mixture, where for clarity in the atomistic region only the tri-glycine is shown. Here the larger cg region serves as a reservoir of water and urea. Figure adapted from Ref. [13]

The atomistic region (indicated as AA or AT in this paper) connects to a coarse-grained region through a transition region (indicated also as hybrid region or as $\varDelta$ region in the paper). The change of resolution would create a discontinuity if the two regions, at different resolution, were directly interfaced. To avoid a sudden discontinuity the transition region is placed between the atomistic and coarse-grained region. This region, whose dimensions are much smaller than the other regions, is artificial and allows for a smooth transition from one resolution to another. In addition it allows to design different schemes, e.g. thermodynamics force/free energy balance, as it will be reported later, to make the physics of the atomistic and coarse-grained region consistent with that of a full atomistic system of reference according to basic principles of statistical mechanics. In the presence of long-range electrostatic interactions, the reaction field method is used to mimic the absence of atomistic charges in the coarse-grained region (see e.g. the case of liquid water [11]). Such a setup can be helpful in various scenarios. Initial ways of looking at AdResS simulations are to interpret it as a magnifying glass applied to coarse-grained simulations. One of the earlier applications, which demonstrated new options based on AdResS, was the study of the solvation shell for a variety of fullerenes of different size [12], where it was shown that the surface-induced structuring of the water does not exceed significantly beyond the second solvation shell. Next, a smaller but still large solute would be a solvated organic molecule in different solvents or solvent mixtures. Figure 2 illustrates a few such cases.

It certainly is beyond the scope of the present work to review all applications of the AdResS concept. After a few basic examples we will focus on work, which points to extensions and new directions. There are several reviews available in the literature, which discuss adaptive resolution methods for coupling different levels of particle based models, path integral quantum and classical models as well particle continuum simulations [6, 14, 15]. Furthermore Ref. [16–18] take a more general soft matter perspective in terms of general multiscale simulations, including adaptive resolution approaches. In the following, the two flavours of AdResS (force based and Hamiltonian based) are discussed within a general framework, followed by some recent applications. For the latter, the focus is on new directions, such as quantum classical coupling, non-equilibrium systems or advanced free energy calculations, which extend the application range of AdResS significantly and show the conceptual equivalence between the two flavours of AdResS.

## Basic idea and new directions

As mentioned before to achieve the equilibrium between a coarse grained and an atomistic region particles/molecules have to move freely without experiencing a barrier between the different regions. This reduces finite size effects in the atomistic region to a minimum and to a very good approximation correctly accounts for fluctuations in a small atomistic region [5, 11, 19]. In the two different regions particles/molecules interact with two different potentials, a microscopic/atomistic one, $H^{AA}$,

1 $$\begin{aligned} H^{\text {AA}}= & {} \sum _{\alpha =1}^N \sum _{i=1}^n \frac{p_{\alpha i}^2}{2 m_{\alpha i}} + V^{\text {int}} + \sum _{\alpha =1}^{N} V_\alpha ^{AA}\nonumber \\ V^{\text {AA}}_\alpha\equiv & {} \frac{1}{2}\sum _{\beta \ne \alpha }^{N} \sum _{ij}^n V^{AA}(|\mathbf{r }_{\alpha i} - \mathbf{r }_{\beta j}|), \end{aligned}$$

and a coarse grained one $H^{\text {cg}}$

2 $$\begin{aligned} H^{\text {cg}}= & {} \sum _{\alpha =1}^N \frac{p_{\alpha }^2}{2 m_{\alpha }} + V^{\text {int}} + \sum _{\alpha =1}^{N} V_\alpha ^{\text {cg}}\nonumber \\ V^{\text {cg}}_\alpha\equiv & {} \frac{1}{2}\sum _{\beta \ne \alpha }^{N} V^{\text {cg}}(|\mathbf{R }_{\alpha } - \mathbf{R }_{\beta }|), \end{aligned}$$

respectively. $p_{\alpha i}$, $m_{\alpha i}$, and $\mathbf{r }_{\alpha i}$ are the momentum, mass, and position, respectively, of atom *i* of molecule $\alpha$.$V^{\text {int}}$ indicates purely intramolecular interactions, for which we do not need to make any assumption and which can be ignored for our current line of arguments. The cg pair potential $V^{\text {cg}}_{\alpha \beta } \equiv V^{\text {cg}}(\mathbf{R }_\alpha -\mathbf{R }_\beta )$ depends on the center of mass (CoM) positions $\mathbf{R }$ of the molecules $\alpha$ and $\beta$ (alternatively e.g. the oxygens in the case of water could be chosen) ; the total CG potential energy of molecule $\alpha$ is thus given by $V^{\text {CG}}_\alpha \equiv \sum _{\beta \ne \alpha } V^{\text {CG}}_{\alpha \beta }/2$. Note that we at this point do not specify any specific property of the coarse grained interaction up to the point that the cg coordinates $R_{\alpha }$ are exactly defined by the atomistic coordinates ${r_{\alpha i}}$. Of course, this also means that the free energy per molecule in the two regions needs not to be the same. Actually to construct a model, where the free energy per molecule considering cg interactions only is the same as with purely atomistic interactions and which satisfies the condition to have the same density and temperature can be quite challenging and in most cases is not practical at all. In the original first setting [5, 6] this put severe constraints on the cg region. The subsequently improved coupling schemes compensate these differences, once molecules move through the transition region, as discussed below.

To move from one region to the other the objects pass through the interpolating transition zone as shown in Fig. 1, which is described by the transition function $\lambda (X)$ (in the one-d case of Fig. 1), determined by the interaction strength originating from either description, namely AA or cg, respectively. So far we only require that $\lambda (X)$ interpolates between 0 and 1 monotonously and that the derivatives at the boundaries of the transition zone are vanishing. Taking X as the cg position of a particle along the transition region there are two coupling schemes, which have been employed in the past, a multiplicative force and an additive energy coupling. The first realizations of AdResS [5] are based on a multiplicative coupling of forces between particles $\alpha$ and $\beta$:

3 $$\begin{aligned} \mathbf{F}_{\alpha \beta }=\lambda (X_\alpha ) \lambda (X_\beta )\mathbf{F}_{\alpha \beta }^{\text {AA}}+[1- \lambda (X_\alpha ) \lambda (X_\beta )]\mathbf{F}_{\alpha \beta }^{\text {cg}}. \end{aligned}$$

This leads to a scheme with well defined forces everywhere in the system, which perfectly satisfy Newton’s 3rd law, $F_{\alpha \beta } = -F_{\beta \alpha }$, even in the transition zone. It, however, comes at the price, that we do not have a well defined Hamiltonian, leading to special deliberations concerning thermodynamic ensembles [15, 20]. This coupling between the two levels of description, however, does not yet account for potential free energy differences between e.g. a cg model of a liquid and the corresponding all atom model at the same density and temperature. As a result they typically have rather different pressures. While the temperature can be kept constant throughout the system via a thermostat, a potential pressure gradient would level off and create a density variation. To compensate for this and keep the density constant everywhere in the system a *thermodynamic force* $\mathbf{F}\text {th} (X)$ is introduced in the transition region, so that the total force acting on particle $\alpha$ reads:

4 $$\begin{aligned}&\mathbf{F}_{\alpha } (X_\alpha )\nonumber \\&\quad =\sum _{\beta ,\beta \ne \alpha } (\lambda (X_\alpha ) \lambda (X_\beta )\mathbf{F}_{\alpha \beta }^{\text {AA}}+[1- \lambda (X_\alpha ) \lambda (X_\beta )]\mathbf{F}_{\alpha \beta }^{\text {cg}})\nonumber \\&\qquad + \mathbf{F}{\text {th}} (X_{\alpha }). \end{aligned}$$

In practice $\mathbf{F}\text {{th}} (X)$, which is zero outside the transition zone, is determined iteratively. Requesting a constant density $\rho$ in the whole simulation box, a good initial choice is $\mathbf{F}{\text {th}_0} (X) = \frac{M}{\rho }\nabla p(X)$, with *M* being the molecular mass. It must be noticed that in the transition region, due to the presence of the thermodynamic force, the linear momentum is not conserved anymore on the atomistic level but is conserved on the fluctuating hydrodynamics level (see footnote 2 on pg.16 of Ref. [15]); this fact has been numerically confirmed in Ref. [21] where, through the use of a DPD thermostat, the conservation of a local linear momentum has been observed. Several applications of this AdResS scheme, which include generalizations and extensions are presented below.

Still, the lack of having a complementary approach, which is based on a Hamiltonian valid in the whole simulation box, can bee seen as a disadvantage, as e.g. a Monte Carlo AdResS is not possible without. Also the discussion of different ensembles would become simpler or more direct. As will be seen below, this comes at the cost that Newton’s 3rd law is not anymore exactly fulfilled everywhere. The Hamiltonian for an energy based AdResS, called H-AdResS [9, 10, 22], is given by

5 $$\begin{aligned} H =\sum _{\alpha i} \frac{p_{\alpha i}^2}{2 m_{\alpha i}} + \sum _{\alpha } \left\{ \lambda _\alpha V^{\text {AA}}_\alpha + (1 - \lambda _\alpha ) V^{\text {CG}}_\alpha \right\} + V^{\text {int}},\nonumber \\ \end{aligned}$$

and contains already some resemblance of the Kirkwood thermodynamic integration. Again, since $V^{\text {int}}$ only describes intramolecular contributions, we do not have to specify this further. This energy function is well defined in the whole simulation box and leads to the overall force $\mathbf{F }_{\alpha i}$ on atom *i* of particle $\alpha$:

6 $$\begin{aligned} \mathbf{F }_{\alpha i}= & {} \sum _{\beta ,\beta \ne \alpha } \left\{ \frac{\lambda _\alpha + \lambda _\beta }{2} \sum _{j=1}^n\mathbf{F }^{\text {AA}}_{\alpha i|\beta j} + \left( 1 - \frac{\lambda _\alpha + \lambda _\beta }{2}\right) \mathbf{F }^{\text {CG}}_{\alpha i|\beta } \right\} \nonumber \\&\qquad + \mathbf{F }^{\text {int}}_{\alpha i} - \left[ V^{\text {AA}}_\alpha - V^{\text {CG}}_\alpha \right] \nabla _{\alpha i}\lambda _\alpha . \end{aligned}$$

Thus forces and energies are well defined in the whole system. Unlike the force based approach there is an explicit drift term $\mathbf{F}^{\text {dr}} = - [ V^{\text {AA}}_\alpha - V^{\text {CG}}_\alpha ] \nabla _{\alpha i}\lambda _\alpha$ like an external field, which accounts for the free energy differences in the AA and CG regions. This in average corresponds to the pressure gradient $\nabla p = \rho < \mathbf{F}^{\text {dr}}>$, eventually leading to an equilibrium between the AA and CG region not necessarily at the anticipated state point. At the same time this term leads to the above mentioned small violation of Newton’s 3rd law fluctuating in time, as the pair forces explicitly depend on the particle positions and not only on their relative distances. In practice this violation averages out almost completely, so that the disturbance of the dynamics due to the small fluctuating violation of momentum conservation usually can be neglected [22]. To compensate for that in average, a modified Hamiltonian is introduced

7 $$\begin{aligned}&H^{\text {MIX}}_{\varDelta } = H - \sum _\alpha \varDelta H(\lambda (\mathbf{R }_\alpha )), \end{aligned}$$

constructed in such a way that

8 $$\begin{aligned}&\mathbf{F }^{\varDelta }_{\alpha i} \equiv \frac{\partial \varDelta H(\lambda )}{\partial \lambda }\biggl |_{\lambda _\alpha } \nabla _{\alpha i}\lambda _\alpha \equiv \langle \left[ V^{\text {AA}}_\alpha - V^{\text {CG}}_\alpha \right] \rangle \nabla _{\alpha i}\lambda _\alpha .\nonumber \\ \end{aligned}$$

Though not exact, this to a very good approximation already compensates the drift term. A few iterations are sufficient to produce e.g. a flat density profile as needed for the magnification glass concept introduced before. In turn this drift term can be interpreted as a position dependent Helmholtz free energy integration term, leading to the following expression

9 $$\begin{aligned}&\langle \left[ V^{\text {AA}}_\alpha - V^{\text {CG}}_\alpha \right] \rangle \simeq \frac{1}{N} \left\langle \left[ V^{\text {AA}} - V^{\text {CG}} \right] \right\rangle _{\lambda _\alpha }\nonumber \\&\varDelta H(\lambda _\alpha ) = \frac{1}{N}\int _0^{\lambda _\alpha } d\lambda ' \left\langle \left[ V^{\text {AA}} - V^{\text {CG}} \right] \right\rangle _{\lambda '} =\frac{\varDelta F(\lambda _\alpha )}{N},\nonumber \\ \end{aligned}$$

for the case where the pressure, but not necessarily the density is the same throughout the simulation box, while

10 $$\begin{aligned}&\varDelta H(\lambda _\alpha ) \equiv \varDelta \mu (\lambda _\alpha ) = \frac{\varDelta F(\lambda _\alpha )}{N} + \frac{\varDelta p(\lambda _\alpha )}{\rho }, \end{aligned}$$

corresponds to a homogeneous density everywhere, as e.g. required for the magnification glass setup discussed in the beginning. Actually taking advantage of that led to several new approaches to calculate free energy differences or solvation free energies, as shown below. Furthermore one also can apply Monte Carlo simulations without any further adjustments. So far AdResS only has been formulated for two body interactions. For three-body or higher order interactions, which can be decomposed in two-body terms (like bond bending) the extension is straightforward [23]. For more complicated situations new force/energy distribution schemes have to be developed.

Thus there are two flavours of AdResS at hand, which can be applied as needed, adding to the versatility of adaptive resolution schemes. However, common to both is the fact that particles freely move throughout the system without experiencing any barrier. Focusing on this aspect rather than on the original simulation ansatz lead to different views on such methods, which originally were not that much anticipated.

**Fig. 3:**
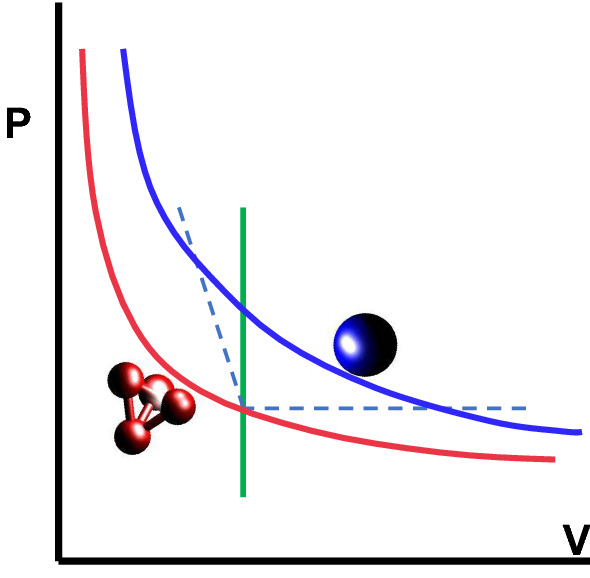
Schematic representation of two EOS for an “ atomistic system” (red) and the “coarse grained” system (blue). The green line indicates the mapping at the same density, leading typically to different, usually higher pressure. As indicated by the broken lines in principle, and as discussed later on, one also could imagine not only a coupling at constant density but to any other point on the EOS of (almost) any coarse grained system

If one looks at the two coupled systems, atomistic and coarse grained, from a statistical mechanics point of view, AdResS and H-AdResS simply couple two different systems. In principle there is no restriction in the way that the density has to be the same etc. as long as one is able to construct a thermodynamic force (AdResS) or a compensation term (H-AdResS) which assures that there is no remaining barrier (actually in some cases this barrier can be explicitly used to calculate the chemical potential [24, 25] or the solvation free energy [26]). Thus these two systems might have quite different equations of state (EOS) as illustrated in Fig. 3. In turn, this provides huge freedom of choice of coupling and gives many interesting options.

Considering the magnification glass point of view, not surprisingly, originally the same density in all regions was requested. As is immediately obvious form Fig. 3, coupling two such systems usually leads to rather different pressures, and usually also to different compressibilities, unless the slope of the two EOS at the same density are the same. The construction of the cg model and the compensation terms in the hybrid region account for that. Water is an extreme example, where an SPCE water model at a pressure of 1 bar and a corresponding density of about $1 \text {g/cm}^3$ and is coupled to a single bead spherical water model. This model, parameterised by a standard structure based coarse graining method, experiences a pressure of about 6000 bar, if the same density and compressibility is required. However both AdResS and H-AdResS robustly can account for that [9, 11]. This original approach, however puts unnecessary constraints on the cg surrounding. Focusing on the properties of the atomistic or more microscopic region the only requirement is the equilibrium between the coupled regions. In other words, as long as we can define an appropriate compensation function in the transition region, we can couple essentially any two different particle based systems (extensions to continuum are discussed below).

This basic concept translates into a high technical flexibility when implementing the coupling code in the simulation algorithm. In the course of the years several coupling techniques have been actually experimented, from an interface pressure corrections [27], to the compensation of the local chemical potential [7], to the more theoretically involved approaches such as thermodynamic force [8], auxiliary Hamiltonian [24] and H-AdResS with the free energy compensation [9]. They all turned out to be robust and delivering consistently satisfactory results. For the systems represented by the EOSs in Fig. 3 this would mean not only coupling systems at positions of the EOSs located exactly above or below each other but to systems at points at rather different places along the EOS. A particularly interesting point is that there are almost no restrictions on the different EOSs themselves which can be coupled and this opens completely new possibilities. Examples, explicitly treated in the next sections, are the coupling of an atomistic liquid to an ideal gas and to non-interacting tracers in a mean-field. Especially the coupling to systems like ideal gas, where everything is known exactly, had been extensively employed in recent work for the efficient calculation of free energy-related quantities [28]. In this context, a clear evidence of the technical flexibility of the root model of AdResS is that the switching function in the transition region can even be removed and as a consequence the force-based and the Hamiltonian-based approach coincide [29, 30]. In such a case the potential of the thermodynamic force becomes equal to compensation term of the Hamiltonian-based coupling [31]. A reservoir represented by an ideal gas or by non-interacting particles in a mean-field, allows for the implementation of a grand canonical scheme in a trivial manner. By that the coarse-grained region can be used as a reservoir of particles of different types at arbitrary thermodynamic conditions to study externally driven atomistic/detailed systems or nonequilibrium situations in cases where the treatment of standard full atomistic systems becomes difficult, some examples are discussed in this paper. Finally, from the formal point of view the treatment of the AT region as an open system [24, 32] led to the construction of general physico-mathematical models that remove the original conceptual limitations [33] and justify both (specific) approaches discussed above on a rigorous formal basis [34, 35]. In this paper, being focused more on the computational aspects, the mathematical analysis will not be discussed in detail, nevertheless it is worth to underline the multidisciplinary character of AdResS. In fact its numerical results have stimulated the formalization of new equations for open particle systems under different conditions [34, 36] which in turn, in perspective, can be automatically embedded in the equations of fluid dynamics [37].

The following chapters describe relevant applications of the method and discuss current technical and conceptual developments which put in perspective potential applications and new directions. While the current paper is dedicated to the AdResS methodology, other similar approaches with their merits and limitations or ways to construct an appropriate coarse grained environment can be found in the following references [38–44].

## Some selected applications of the AdResS method

In this chapter a few applications of the original AdResS setup are presented which go beyond simple homogeneous systems, studied on two different levels of resolution. One area of research, which is especially suited to be studied by AdResS simulations deals with the behavior of large solutes in solvent or solvent mixtures as a function of varying (external) constraints. That can be the change of temperature or composition variations of solvent mixtures [45], where the response to an external stimulus is investigated. In another example a protein, hen egg-white lysozyme, with a binding ligand has been studied, where the all atom region containing solvent (water), the active region of the protein and the ligand are embedded in coarse grained water while the outer parts of the protein were described by an elastic network model [46]. By varying the size of the atomistically described active protein sequence the coupling of ligand binding to the overall conformation fluctuations could be investigated. Other studies look at larger conformational changes or aggregation phenomena, as will be shown in more detail below.

### Self-adjusting atomistic region

**Fig. 4:**
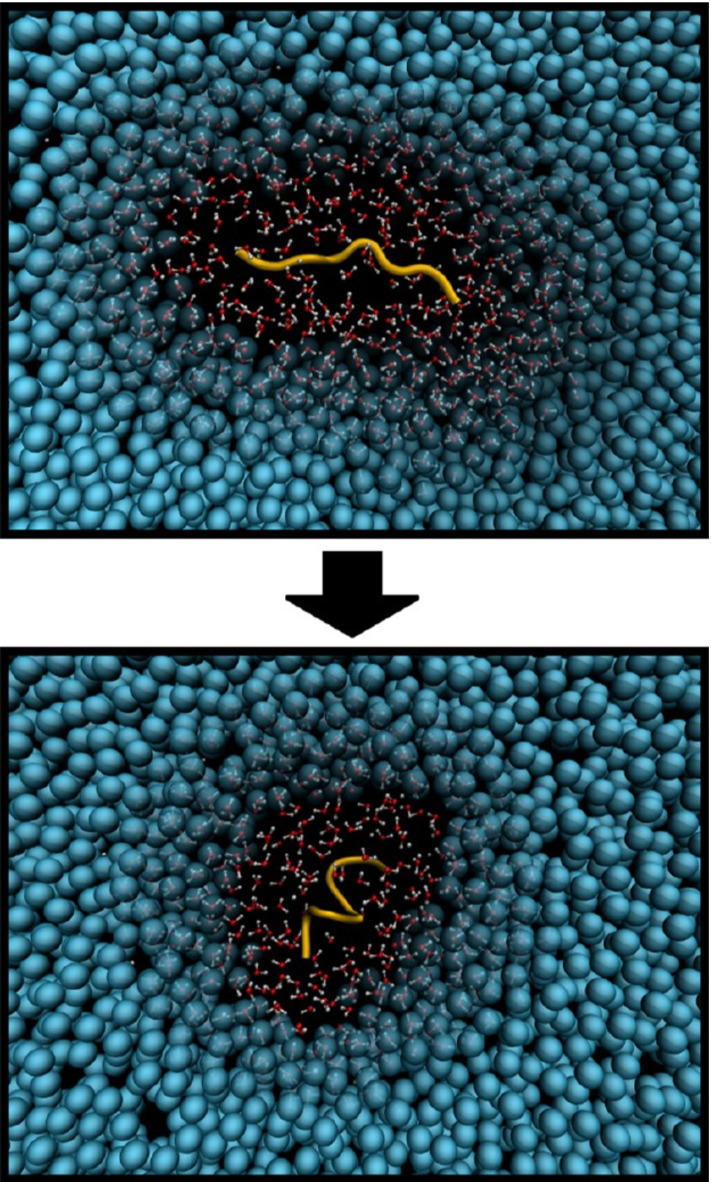
Simulation snapshots showing the folding process of polyalanine 9 in aqueous solution. The atomistic region containing the polypeptide and a layer of water of 1 nm size adjust following the protein conformation. Reprinted with permission from Ref. [47]. Copyright (2016) American Chemical Society

Computer simulations of large (bio-)molecules, such as poly-peptides, require simulation boxes whose linear size is larger than the chain’s radius of gyration. Hence, even a modest increase in the size of the molecule could result in a huge simulation box in which only a relatively small subdomain, the polypeptide and its solvation shell, becomes relevant. In this scenario, the use of the AdResS method might be advantageous, most notably if the atomistic region adjusts to the conformational changes of the protein (Fig. 4).

Such a version of AdResS with a self-adjusting atomistic region has been developed and validated for the study of folding of polyalanine-9 in aqueous solution [47]. The atomistic region is defined by overlapping spheres with centers pinned to atoms in the protein. All the spheres have radius $r_\mathrm{at} + d_{\mathrm{hy}} = 2$ nm with $r_{\mathrm{at}} = 1$ nm. Thus, the polypeptide is always modelled at the atomistic level, including a solvation shell of atomistic water of 1 nm thickness. As a matter of fact, it has been demonstrated that this size of the hybrid region is sufficient to preserve the protein’s solvation properties when compared to the fully-atomistic, reference case [48].

**Fig. 5:**
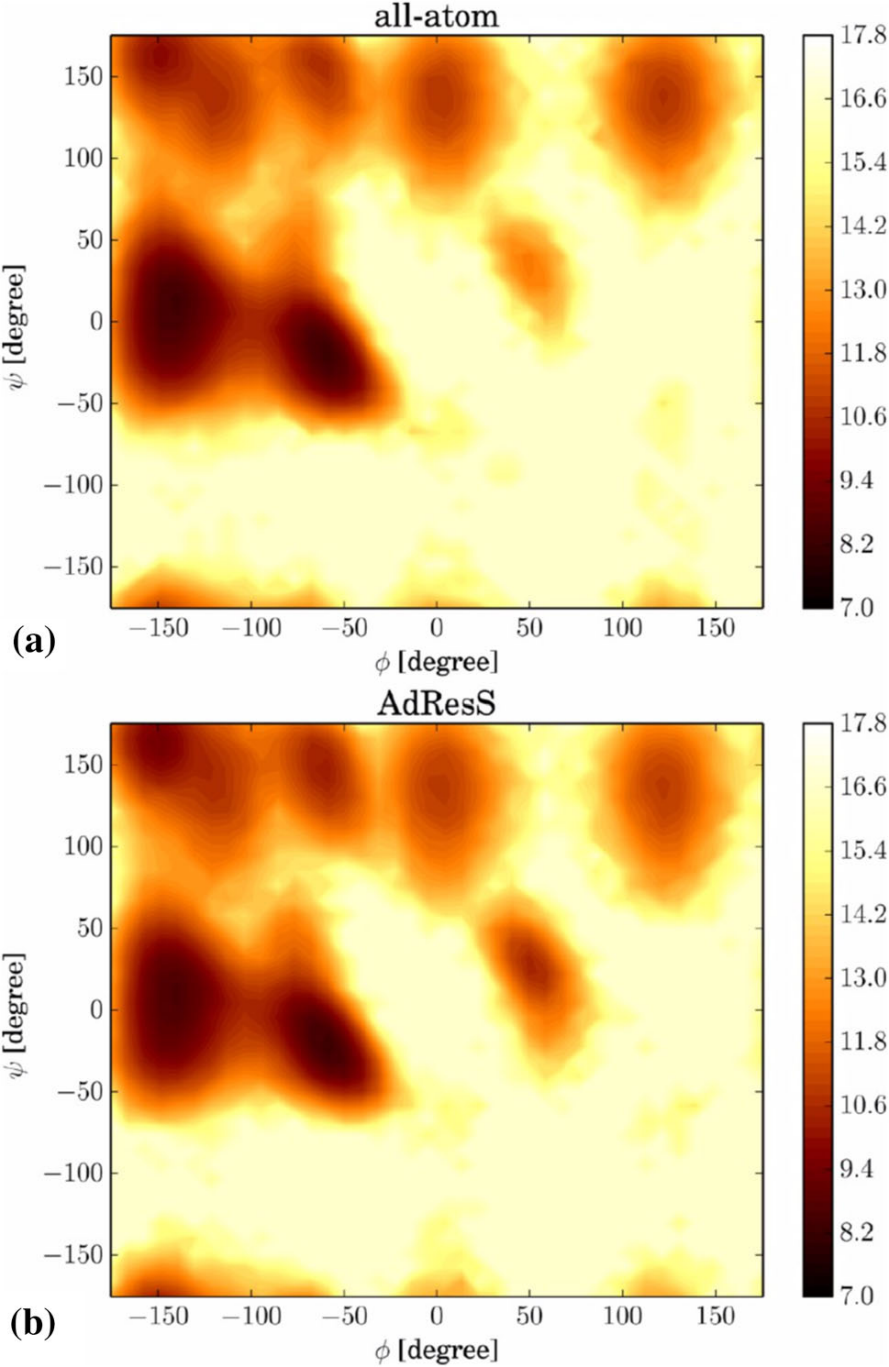
Ramachandran plots showing the negative logarithm of the probability density distribution for the **a** fully-atomistic and **b** adaptive resolution simulations of the folded polypeptide. Reprinted with permission from Ref. [47]. Copyright (2016) American Chemical Society

To ensure consistency between all-atom and AdResS computations one has to verify that the peptide folds to the same region of conformational space in both cases. To this aim, among others, Ramachandran plots (Fig. 5) showing the probability density distribution of the backbone dihedral angles $\phi$ (about the N-C$_{\alpha }$ bond) and $\psi$ (about the C$_\alpha$-C bond) calculated for the folded peptide in fully-atomistic (a) and AdResS (b) simulations have been analysed. It is apparent from the figure that both calculations give within statistical error an identical landscape, in which a prominent formation of $\alpha$-helix structures ($-180^{\circ }< \phi < {0}^{\circ }$ and $-{100}^{\circ }< \psi < {45}^{\circ }$) is observed.

### Aggregation of micelles in water: taking advantage of locality of interactions

**Fig. 6:**
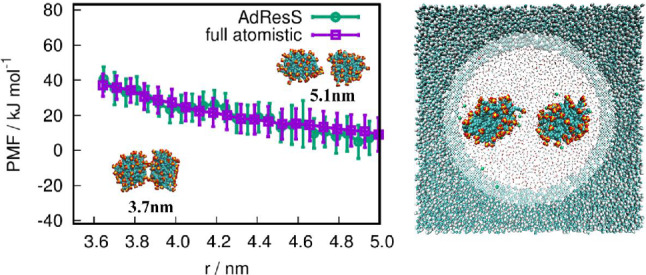
Free energy of aggregation of micelles in water. The AdResS simulation agrees very well with the full atomistic simulation of reference. The study through the AdResS approach allows for a sizable saving of computational resources. Figure reproduced from Ref. [49], Copyright Wiley-VCH Verlag GmbH and Co. KGaA. Reproduced with permission

For the above example the diameter of the atomistic region was chosen to be on the safe side, meaning that effects of structural details of the surrounding solvent beyond this diameter are irrelevant for the solvation properties of the solute. In turn one can vary the diameter of the atomistic region, testing the range at which such effects are relevant. This question first has been addressed in the context of fullerenes (cf. Figure 2) [12] and a small protein, ubuquitin [48], both in water. Here this concept is extended to the interaction of micelles in water as illustrated in Fig. 6. AdResS is used to calculate the free energy/potential of mean force during the aggregation process of such large solutes in water. Water molecules surrounding a solute are characterized by structural and dynamical properties which substantially differ from those of bulk water. In particular, in the aggregation process of two micelles, as in Fig. 6, the extension of water-mediated effects in space is relevant. Such water-mediated effects require the explicit description of the hydrogen bond network which, in turn, needs to be treated at atomistic level. The AdResS approach, by construction, can determine the minimum size of the solvation region around each solute where an explicit atomistic resolution is mandatory (see also Ref. [12]): if all considered physical quantities calculated in a molecular high resolution spherical domain around the solute in AdResS agree with the equivalent quantities calculated in a reference full atomistic simulation, then the atomistic degrees of freedom outside the atomistic domain are not required and can be neglected. As a consequence, one can determine the minimum size of the atomistically described solvation region. This means that the water-mediated effects in the interaction of two micelles only become relevant when the two minimal solvation shells (of the size determined by AdResS) get in contact. AdResS in such a case brings a twofold computational advantage: (1) it requires a calculation for a single micelle (i.e. a small system) for determining the size of the solvation shell. Such a study determines the minimal distance of relevance in the micelle-micelle aggregation process. As a consequence, in a two-micelles calculation, distances larger than the minimal one can be excluded *a priori* and reduce in a sizable manner the computational costs of the study; (2) once the minimal distance is defined, then one has got the usual gain of AdResS for studying the two-micelles system. The results of AdResS agree very well with a reference full atomistic simulation and show the expected significant reduction of computational costs (see Ref. [49]). Similar situations involving spatial locality occur for ionic liquids [50, 51]. Through AdResS one can identify a local behavior, and for modeling purposes that lead to computational saving. In this specific case it is worth to notice that surprisingly the expected long range effects of the Coulomb interactions can be shown, with AdResS, to not be relevant. This result confirms the conclusions of other theoretical studies and of experiments [52]. A further selection of representative applications of AdResS in the context of local properties can be found in [53].

### Equation of state (EoS) of high density DNA arrays

**Fig. 7:**
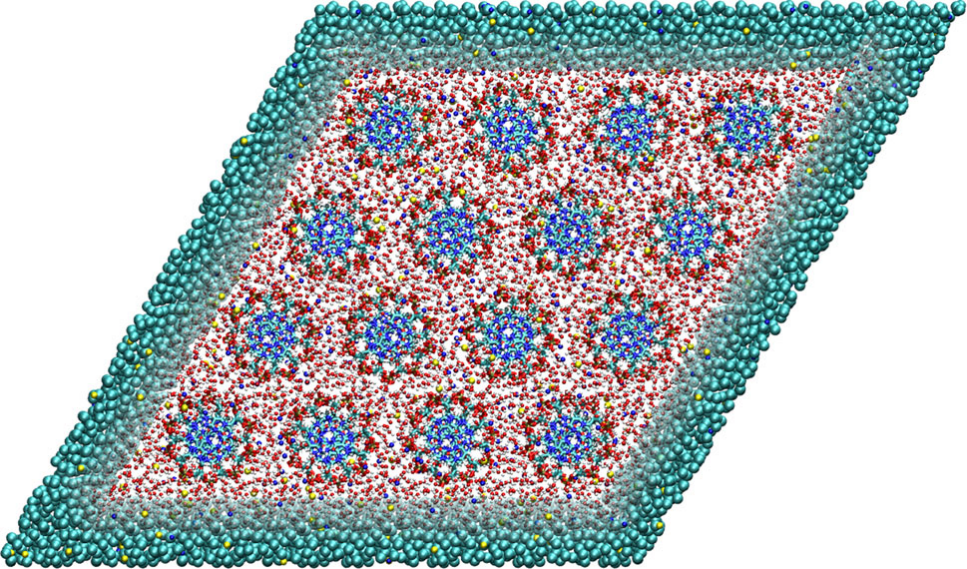
Top-down view on a system of solvated DNA molecules arranged on a hexagonal lattice. The DNA molecules are described with the atomistic model whereas the salt solution is modeled with the atomistic resolution inside the rhombic region with the DNA molecules and the coarse-grained representation outside. Figure adapted and reproduced from Refs. [54, 55]

As examples described in previous sections illustrate, AdResS can be directly employed for simulation studies of rather complex biomolecular systems. Dense DNA in the columnar phase that one encounters in vivo e.g. in viral capsids or eukaryotic nuclei is another such example. To properly describe the physical properties of high density DNA arrays and their EoS simulations should correctly define and maintain the osmotic isobaric ensemble, equivalent to the solvent grand-canonical ensemble that allows for the exchange of ions and water with the environment [54]. To this end, the whole atomistic DNA array is embedded within a reservoir of coarse-grained water molecules and ions [55] as depicted in Fig. 7. The DNA array and reservoir are separated by a semi-permeable membrane that allows the water molecules and ions (but not the DNA molecules) to freely pass between the two domains. The osmotic pressure of the DNA array is then determined by measuring the force exerted by the DNA subphase on the confining membrane. The force is computed via a repulsive interaction between the semipermeable membrane and DNA backbone. By varying the DNA density, local packing symmetry, and counterion type, the osmotic EoS has been obtained during with the hexagonal-orthorhombic phase transition, together with the full structural characterization of the DNA subphase in terms of its positional and angular orientational fluctuations, counterion distributions, and the solvent local dielectric response profile [55]. However, to perform truly open grand-canonical molecular simulations one should resort to the open boundary molecular dynamics (OBMD) that permits simulations directly in the grand-canonical ensemble and is discussed in Sect. 4.3.3.

### Beyond one to one mapping: supramolecular coarse-grained models

**Fig. 8:**
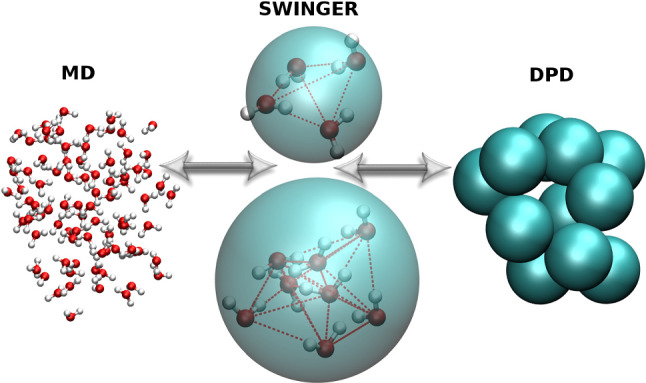
Concurrent coupling of atomistic and supramolecular DPD water using AdResS augmented by SWINGER. Figure reproduced with permission from Ref. [21], Copyright 2017, AIP Publishing

In water, the most common solvent in biomolecular systems, the average lifetime of tetrahedral clusters due to hydrogen bonding is on a picosecond time scale. Thus it is tempting to use this to construct a coarse grained water model, which keeps the all atom clusters once the atomistic region is left. This, however, represents a major challenge for concurrent coupling of atomistic and supramolecular water models in biomolecular simulations. Not only clusters have to be identified but also water molecules have to be correctly distributed to the cg sites, without leaving single molecules behind. To account for this the clustering algorithm SWINGER [21, 56, 57] has been developed, which dynamically creates, breaks and remakes clusters of water molecules. To do this consistently, the number of molecules in a cluster has to be exactly equal to the applied atomistic-to-supramolecular mapping. Furthermore the clustering should be optimized in terms of minimal distances of molecules within the clusters. The frequency of the algorithm’s initialization should be on a picosecond timescale, and the algorithm should leave the coordinates and velocities of atoms intact. In combination with AdResS, this then allows to seamlessly couple atomistic and coarse-grained force fields, in which one coarse-grained bead represents several atomistic molecules (four in the case of the MARTINI force field [58]). In this way, at each given time, it is exactly known which water molecules belong to the corresponding coarse-grained bead. The algorithm, however, which essentially is a very efficient sorting method, is not linked to a specific interaction. It thus can also be used for coupling MD with a Dissipative Particle Dynamics (DPD) [59, 60] for example. This concurrent coupling [21], which bridges atomistic and mesoscopic hydrodynamics, is schematically depicted in Fig. 8. Other authors have coupled a particle based MD simulation within an AdResS setup to a multi particle collision (MPC) scheme in order to account for a hydrodynamic coupling [61].

## Open system statistical mechanics and applications of different coupling approaches

As discussed in Sect. 2, the root model of AdResS allows for different coupling protocols between the AT region and the coarse-graining region. The interpolation of the atomistic and coarse-grained potentials in H-AdResS leads to a free energy compensation scheme which properly balances the missing free energy contributions of the AT region due to loss of degrees of freedom in the coarse-grained region equally for both, MD and MC simulations. The force interpolation in AdResS leads to a thermodynamic force which automatically fixes the thermodynamic state point in the AT region. The two protocols produce, within numerical error bars, the same results compared to each other and to corresponding full atomistic simulations. In fact they can be seen as different technical expressions of the same balancing process: the chemical potential over the whole AdResS box is uniformed to the value of the chemical potential of the full atomistic simulation of reference [24, 25, 28]. The process of balancing the chemical potential so that the AT region and the CG region have the same value, suggests that the AT region can be seen as an open system, a Grand Canonical-like/Grand Ensemble, in contact with a reservoir of coarse-grained particles through a coupling region. Actually as we make the AT region much larger than $\varDelta$ and the CG region much larger than the AT region, we should observe more and more a Grand-Canonical behaviour of the AT region as the size of the AT region increases. This idea was explored in the so-called GC-AdResS formulation of the method [32]; the corresponding detailed discussion is reported in the next subsection. For the case of coupling to non-interacting particles, where a truly grand canonical algorithm easily can be implemented, this even does not require a large CG region [30, 62].

### Models of open system simulations for the AT region

#### Role of external potential and density for H-AdResS

Gaining a computational advantage by replacing the coarse-grained representation by a reservoir of non-interacting particles (ideal gas) is somewhat obvious [62]. More importantly, it highlights the identification of the compensating function as the excess chemical potential of the atomistic system compared to a system, where everything is known exactly. Indeed, calculating the chemical potential in this way is equivalent to a spatially-resolved thermodynamic integration (SPARTIAN) [28]. Moreover, the ideal gas can easily be treated as an open reservoir such that the whole simulation setup samples a truly grand canonical ensemble [63]. To sum up, the coupling to an ideal gas allows us to simulate an inhomogeneous, open system at a constant chemical potential.

In particular, we can write the H-AdResS Hamiltonian (Eq. 5) with the correction term of Eqs. 9, 10 for a fluid composed by $\langle N\rangle$ molecules, in contact with an infinite ideal gas reservoir, as

11 $$\begin{aligned} H_{[\lambda ]}(r,p) = {\mathcal {K}} + V^\mathrm{intra} + \sum _{\alpha =1}^{\langle N\rangle }\{ \lambda _{\alpha }V_{\alpha } + V^\mathrm{ext}(\lambda _{\alpha })\},\nonumber \\ \end{aligned}$$

(*r*, *p*) are positions and momenta and ${\mathcal {K}}$ the total kinetic energy of the system, respectively. The term $V^\mathrm{intra}$ describes intra-molecular interactions. The intermolecular interactions are included in the term $V_{\alpha }$. As before, the switching field determines the molecules’ identity, with $\lambda _{\alpha }\equiv \lambda ({\mathbf {R}}_{\alpha })$ and ${\mathbf {R}}_{\alpha }$ the position of the center of mass of the molecule $\alpha$. When $\lambda =0$ the Hamiltonian describes a homogeneous ideal gas system provided $V^\mathrm{ext}(0) = \mathrm{constant}$, which we can set as $V^\mathrm{ext}(0) = 0$. As anticipated, this Hamiltonian describes an open inhomogeneous system, namely, an interacting system, under the influence of an external field, embedded in an infinite reservoir.

This interpretation allows us to use the classical density functional theory [64–66] (DFT) to investigate the connection between the external potential and the corresponding equilibrium density. Recently, Baptista et al. [35] have shown that the grand potential $\Omega _{[\lambda ]}$, as obtained from the Hamiltonian in Eq. (11), can be written as a functional of the system’s density $\rho ^{[\lambda ]}({\mathbf {r}})$

12 $$\begin{aligned}&\Omega _{[\lambda ]}[\rho ^{[\lambda ]}({\mathbf {r}})] \nonumber \\&\quad =F_{[\lambda ]}[\rho ^{[\lambda ]}({\mathbf {r}})] + \int d{\mathbf {r}}\, \rho ^{[\lambda ]}({\mathbf {r}})(V^\mathrm{ext}(\lambda ({\mathbf {r}})) - \mu (\lambda ({\mathbf {r}}))),\nonumber \\ \end{aligned}$$

where $F_{[\lambda ]}$ is the Helmholtz free energy and $\mu$ the chemical potential of the system. This functional Legendre transform connects the Helmholtz free energy with the grand potential that, being a functional of the density, represents the cost in free energy necessary to fix the system’s density at $\rho ^{[\lambda ]}({\mathbf {r}})$. We thus find the density field $\rho _{0}^{[\lambda ]}$ that minimises this cost by evaluating the functional derivative

13 $$\begin{aligned} \left. \frac{\delta \Omega _{[\lambda ]}[\rho ^{[\lambda ]}]}{\delta \rho ^{[\lambda ]}}\right| _{\rho ^{[\lambda ]} = \rho _{0}^{[\lambda ]}} = 0\, . \end{aligned}$$

Finally, to ensure thermodynamic consistency in the adaptive resolution framework, we assume the Helmholtz free energy being independent of the switching field $\lambda$. This implies that

14 $$\begin{aligned} F_{[\lambda ]}[\rho ^{[\lambda ]}] = F^\mathrm{id}[\rho ({\mathbf {r}})]\, , \end{aligned}$$

with $F^\mathrm{id}=\beta ^{-1}\int d{\mathbf {r}}\rho ({\mathbf {r}})\{\ln (\lambda ^3_{T}\rho ({\mathbf {r}})) - 1\}$ the Helmholtz free energy of the ideal gas (the reference state at $\lambda =0$), $\rho ^{[0]}({\mathbf {r}})=\rho ({\mathbf {r}})$ and $\lambda _{T}= (\hbar ^2\beta /2\pi m )^{1/2}$ the thermal, de Broglie, wavelength.

By including this condition into the minimisation condition (Eq. 13), we arrive at an expression for the density field in terms of the external potential and the excess chemical potential $\mu ^\mathrm{exc}$

15 $$\begin{aligned} \rho ({\mathbf {r}}) = \rho _{0}\exp {(-\beta \{ V^\mathrm{ext}(\lambda ({\mathbf {r}})) - \mu ^\mathrm{exc}(\lambda ({\mathbf {r}})) \})}\, . \end{aligned}$$

In particular, a constant density profile $\rho _0$ is obtained when

16 $$\begin{aligned} V^\mathrm{ext}(\lambda ({\mathbf {r}})) = \mu ^\mathrm{exc}(\lambda ({\mathbf {r}}))\, . \end{aligned}$$

The analogy with DFT guarantees a one-to-one correspondence between the external potential and the corresponding equilibrium density. In contrast to standard DFT, where the external potential is known and the density is the target quantity, in adaptive resolution simulations we typically fix the reference density to evaluate the external potential. As so far discussed in the context of H-AdREsS these arguments can applied to both MD and MC simulations. More importantly, this interpretation of the adaptive resolution method motivates the investigation of various atomistic/ideal gas interfaces that have not been systematically investigated before. In particular, in the context of open systems, we can modify Eq. (14) to investigate diverse out-of-equilibrium conditions.

#### Liouville equation and GC-AdResS

The possibility of treating the AT region of AdResS as an open system requires a reformulation of some basic concepts of particle dynamics. In fact a Liouvillian description, as usually assumed in standard Molecular Dynamics of closed systems [67], is no more possible and the existence of a Liouvillian-like operator/time-propagator for open systems needs to be proven. This is not a mere formal request without any practical consequence. In fact a physically valid/first principles definition of, e.g., time correlation functions implies the existence of a Liouvillian operator/time-propagator [15, 68]; without that any technical protocol of Molecular Dynamics which calculates time correlation functions for open systems must be considered empirical. The need of a solid mathematical physics based model of open systems that can serve as a conceptual reference of AdResS, led some of us to consider the so-called Bergmann-Lebowitz model of open systems [69–71] and to map its guiding principles onto the technical features of AdResS.

Bergmann and Lebowitz (BL) derived a generalization of Liouville’s equation for systems with open boundaries [69, 70]. The interaction between the system and the reservoir(s) creates a discontinuous transition in the former from an initial state with *N* particles ($X^{'}_{N}$) to a new state with *M* particles ($X_{M}$), while the reservoir is not influenced by the exchange. The contingent probability of transition from one state to another is expressed by: $K_{NM}(X^{'}_{N},X_{M})dX^{'}dt$ where the the kernel $K_{NM}(X^{'}_{N},X_{M})$ is a stochastic function, independent of time, which expresses the probability per unit time that the system, upon interaction with the reservoir(s), makes a transition from $X_{M}$ to $X^{'}_{N}$.

The term:

$$\begin{aligned}&\sum _{N=0}^{\infty }\int dX^{'}_{N}[K_{MN}(X_{M},X^{'}_{N})\rho (X^{'}_{N},N,t)\\&\quad -K_{NM}(X^{'}_{N},X_{M})\rho (X_{M},M,t)], \end{aligned}$$

formalizes the total system-reservoir interaction which leads to the general equation of time evolution for the probability in phase space:

17 $$\begin{aligned} \frac{\partial \rho (X_{M},M,t)}{\partial t}= & {} -\{\rho (X_{M},M,t),H(X_{M})\}\nonumber \\&+\sum _{N=0}^{\infty }\int dX^{'}_{N}[K_{MN}(X_{M},X^{'}_{N})\rho (X^{'}_{N},N,t)\nonumber \\&-K_{NM}(X^{'}_{N},X_{M})\rho (X_{M},M,t)]. \end{aligned}$$

Here $\rho (X_{N},N)$ is the probability distribution of the system in phase space for the realization of *N* particles, $H(X_{M})$ is the Hamiltonian of the system corresponding to the point $X_{M}$ and $\{*,*\}$ are the standard Poisson brackets. The extension to more reservoirs can be expressed by adding them on the r.h.s of Eq. 17.

In equilibrium, from the condition of flux balance, that is the integral on the r.h.s of Eq. 17 equal zero, follows that the stationary solution of Eq. 17 is a Grand Ensemble corresponding to the Grand Canonical ensemble with: $\rho _{M}(X_{M},M)=\frac{1}{Q}e^{-\frac{1}{k_{B}T} H_{M}(X_{M})+ {\frac{\mu }{k_{B}T} M}}$ where $k_{B}$ is the Boltzmann constant, *T*, the temperature and $\mu$ is the chemical potential. This is a necessary and sufficient condition for stationarity with respect to the Grand Canonical distribution [70, 71].

AdResS can be interpreted as a dynamic-like approximation of the BL stochastic process [72] thus the claim that its atomistic region follows a grand canonical-like statistics is rigorously justified. In addition, numerical simulations have shown that indeed the AdResS protocol reproduces typical Grand Canonical features for the atomistic region when compared with the results of a subsystem of a large full atomistic simulation of reference [24, 32]. For example, in the thermodynamic limit the isothermal compressibility, $\kappa _{T}$, in a Grand Canonical ensemble, is related to the fluctuations of the particle number by [73]: $\rho k_{B}T\kappa _{T}=\frac{\langle N^{2} \rangle -\langle N \rangle ^{2}}{\langle N\rangle }$, with $\rho$ the density of particles. The numerical test performed with AdResS considered 100,000 water molecules out of which about 20,000 in the AT region at ambient conditions. Results show that AdResS reproduces the expected compressibility. Further tests of the energy fluctuation and its covariance in the AT region confirm the predicted behavior. The AdResS protocol based on these tests is labeled GC-AdResS. The conditions regarding the size of the simulation would suggest that a Grand Canonical interpretation of the AT region of AdResS is possible only for very large systems/reservoirs, thus it would have as a price a loss of numerical efficiency. However, the analogy to the BL model allows to simplify the AdResS structure further and reduce it to few essential requirements, thus to an efficient numerical tool. The problem of the size of CG region can be avoided by introducing a true grand canonical ensemble, if the CG interactions allow for an efficient particle implementation scheme as illustrated below.

### Employing open system AdResS simulations

#### AT region as an open system embedded in a reservoir of non-interacting particles

**Fig. 9:**
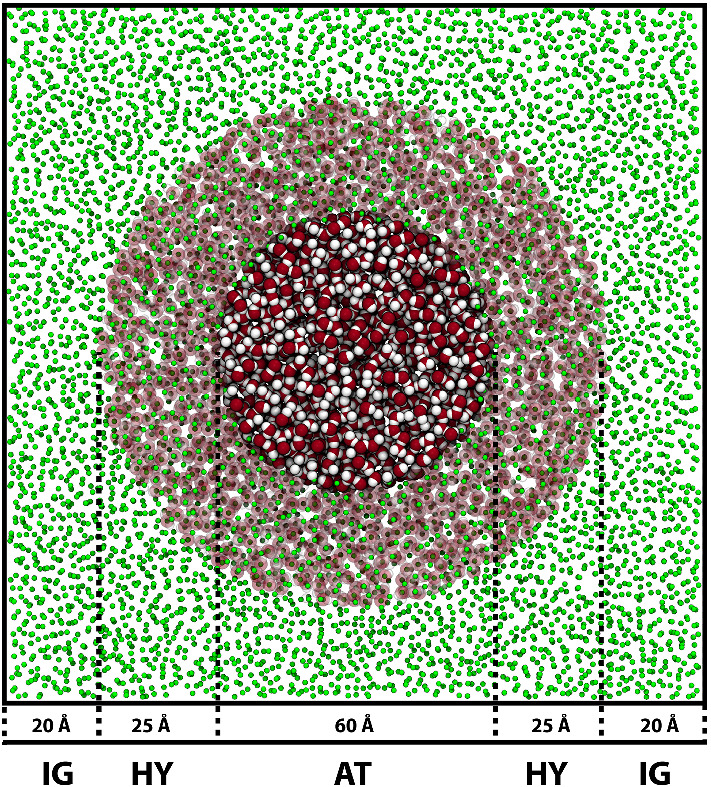
Simulation snapshot showing a typical AdResS configuration representing atomistic water embedded into a reservoir of thermalised, non-interacting particles. A spherical atomistic (AT) region or radius 30 Å  is embedded into a simulation box of linear size of 150 Å. The hybrid (HY) region is a spherical shell of maximum thickness 25 Å, and the ideal gas (IG) occupies the remaining free space. Figure taken from Ref. [35], licensed under a Creative Commons Attribution (4.0) license

As discussed above, in the AdResS framework it is possible to consider the AT system as an open system embedded in a particle reservoir whose mainly purpose is to allow the exchange of particles within the system. In this context, the type of intermolecular potential used for the particles in the reservoir becomes irrelevant. Indeed, this has been applied by treating particles present in the reservoir at the ideal gas level [62] (Fig. 9).

**Fig. 10:**
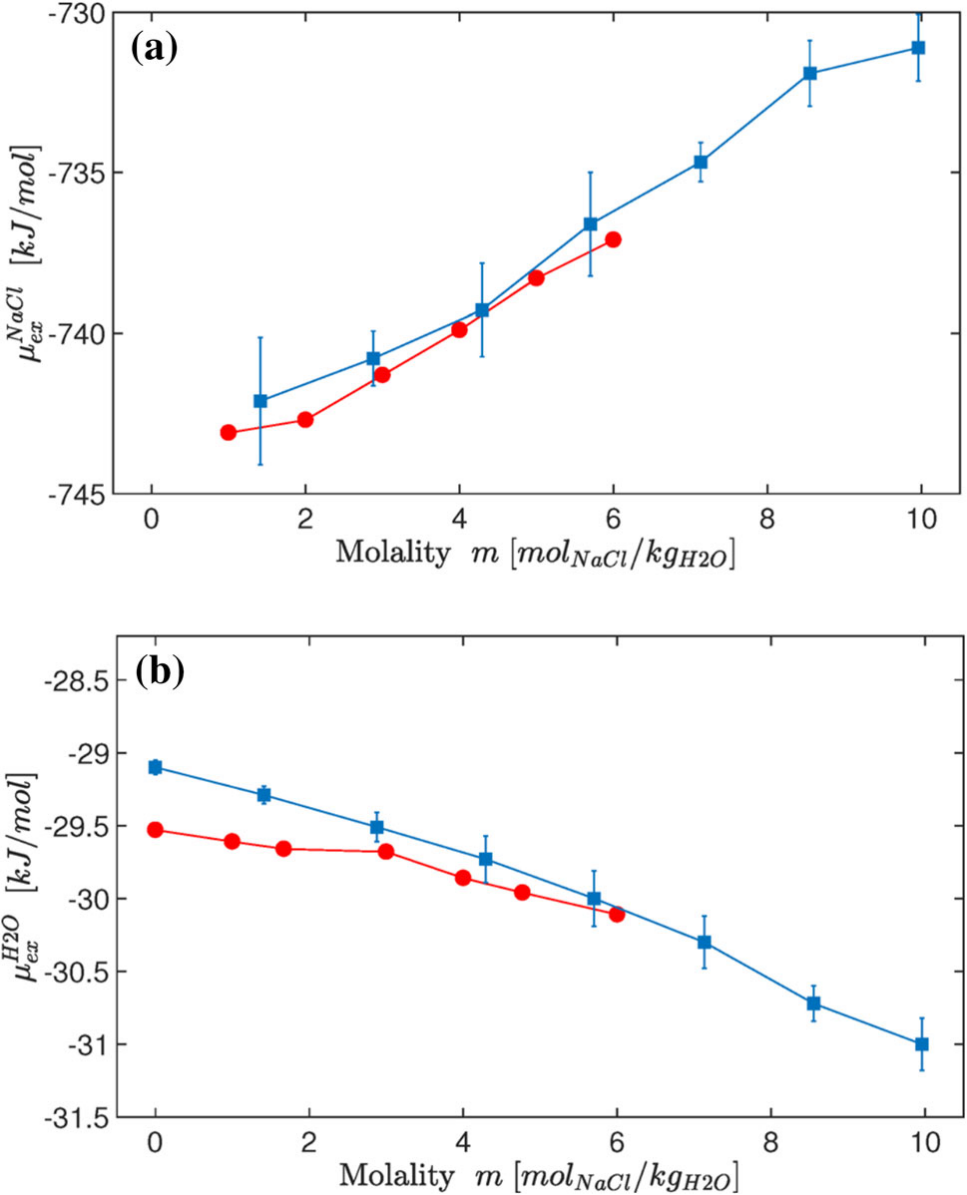
Excess chemical potential of molecular NaCl $\mu _\mathrm{exc}^\mathrm{NaCl}$ (**a**) and water $\mu _{\mathrm{exc}}^{{\mathrm{H}}_2{\mathrm{O}}}$ (**b**) as computed for different salt concentrations. The results obtained with the AdResS and BAR [74] methods are represented by blue squares and red circles, respectively. Reprinted with permission from Ref. [28]. Copyright (2018) American Chemical Society

In such a case, the most direct approach is to enforce a constant density profile across the simulation box. It has been explicitly demonstrated in the context of AdResS that the integral of the external force applied to ensure a uniform density across the system equals the difference in chemical potential between the two resolutions [25]. In the context of H-AdResS this algorithm is dubbed as SPARTIAN method. By coupling to an ideal gas reservoir, the excess chemical potential is automatically obtained [28]. We present here an example of such a calculation for prototypical aqueous solutions of sodium chloride. Results of this procedure agree well with existing computations obtained with particle-insertion based methods (Fig. 10). However, they cover a larger concentration range of sodium chloride. In addition to its practical value, this result contains a fundamental idea. A reference density, imposed throughout the simulation box, allows us to perform molecular dynamics simulations for an open system at constant chemical potential. Moreover, as we will see in Sect. 4.2.2, it is straightforward to implement a particle insertion protocol in the ideal gas reservoir such that an infinite reservoir is explicitly taken into account [63].

#### Particle insertion/deletion protocol

The coupling between atomistic and ideal gas regions at a constant chemical potential opens the possibility to sample the grand canonical ensemble. Indeed, at temperature *T*, volume *V* and chemical potential $\mu$ the probability that the ideal gas reservoir has exactly *N* particles follows a Poisson distribution. This condition allows us to devise a Metropolis algorithm in which the target number density $\rho$ increases/decreases by a small amount $\nu$ with probability given by [63]

$$\begin{aligned} \text {acc}(\rho \rightarrow \rho \pm \nu )=\text {min}[1,\text {exp}(-k_{\mu }\nu (\nu \pm 2(\rho -\rho ^{*})))]\, , \end{aligned}$$

with $\rho ^*$ the target density and $k_{\mu }$ a free parameter. This simple procedure guarantees that the system is effectively in contact with an infinite particle reservoir. More importantly, with the appropriate geometry of the simulation box, different target densities can be simultaneously imposed to investigate non-equilibrium conditions.

### Complementary coupling approaches and applications

#### AT region as an open system embedded in a mean-field particle reservoir

**Fig. 11:**
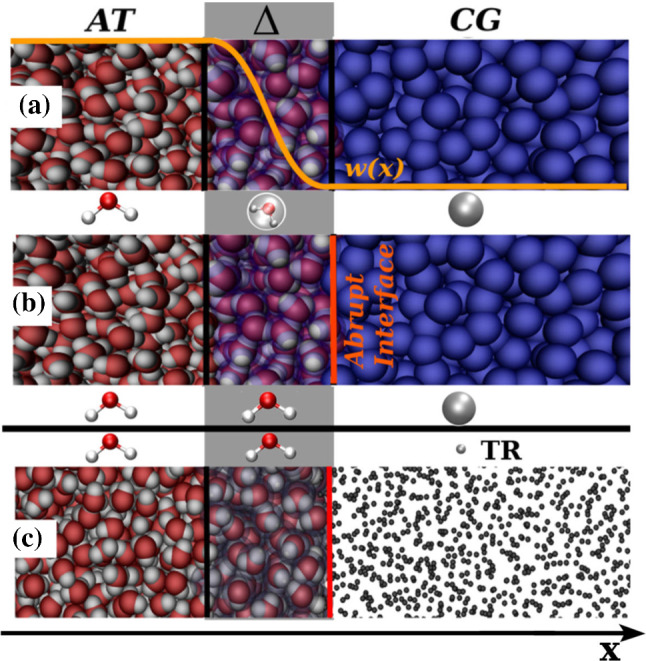
**a** Pictorial representation of the original idea of AdResS;  **b** Abrupt version of AdResS as in Ref. [29] i.e. in the $\varDelta$ region a switching function is no more required. Atomistic molecules interact with coarse-grained molecules (and vice versa) via a coarse-grained potential acting on the center of mass of each molecule.  **c** The CG region becomes a TR region, that is a region of non-interacting point-particles (tracers), the thermodynamic force is calculated over the whole $\varDelta \cup \mathrm {TR}$ region and a capping force avoids clashes of atoms upon acquisition of atomistic degrees of freedom by tracers entering in the $\varDelta$ region at unphysical distances from each other. Figure reproduced from Ref. [30]

The AdResS set up based on the concept of Sect. 4.1.2, automatically represents a well founded physical model of open systems. The rather general conditions suggest any simulation fulfilling them would lead to a proper physical description of the AT region. Based on this a first technical step further to simplify the AdResS set up was the modification of the coupling region with the removal of the interpolation function $\lambda (x)$ and the construction of an abrupt interface between the region at atomistic and the coarse-grained resolution, respectively. The $\varDelta$ region now is a region at atomistic resolution where molecules interact with atomistic interactions with the AT and within the $\varDelta$ region, but interact with coarse-grained interactions with the molecules located in the coarse-grained region (see panel (b) in Fig. 11).The thermodynamic force is calculated in $\varDelta$ as before. This set up has successfully been tested an implemented for liquid water, and for the more challenging case of ionic liquids [29]. Its advantage lies in the significant simplification of the implementation of the algorithm, thus making it easily transferable to other simulation codes. A further aspect, worth a note is that by introducing an abrupt coupling, the force-based AdResS and the Hamiltonian-based AdResS become equivalent, since there is no more an interpolation of quantities via $\lambda (x)$. In fact, building on the results of Refs. [62, 75] the coarse-grained region in the abrupt coupling can be reduced to a system of non-interacting point-particles (tracers) and the calculation of the thermodynamic force is extended to the tracers region as well. The resulting total interaction potential in this set up reads:

$$\begin{aligned} U_\mathrm {tot}= U_\mathrm {tot}^\mathrm {AT} + \sum _{k\in \varDelta \cup \mathrm {TR}}\phi (X_k), \end{aligned}$$

where $U_\mathrm {tot}^\mathrm {AT}$ is the total potential energy due to atomistic force fields in the AT region and the *k*-sum runs over all molecules in $\varDelta$ and all tracers [30] (see also panel (c) Fig. 11). Numerical instabilities are avoided by a force capping in the $\varDelta$ region, i.e. by truncating each Cartesian component of the total force vector at a prescribed maximum value. The capping force is applied to molecules which, upon entering the atomistic region, are getting too close to each other. It turns out that the total number of capping events per time step only affects a small fraction (around $1\%$) of the total number of molecule-molecule interactions in the $\varDelta$ region and affects the AT region not in a relevant way. It is remarkable that the combined action of the capping force, the thermodynamic force and the thermostat is sufficient to avoid numerical instabilities due to artificially large or discontinuous forces in the $\varDelta$ region without producing any artifact for the atomistic region. Since the action of the neighborhood on each tracer depends only on the position of the tracer in space, independent of the global tracer configuration, each tracer experiences $\phi (x)$ and a thermostat (to fix the temperature in TR) as an effective mean field. Thus the AT region is embedded effectively in a mean field particle-based reservoir. A first application is presented in the following chapter on membrane solvation. A MC scheme to change coupling abruptly was put also forward by Abrams [76]. The key difference with the current approach is the absence of a transition region characterized by a thermodynamic force derived in a systematic manner.

#### Study of the hydration shell of a membrane with the open system protocol of AdResS

**Fig. 12:**
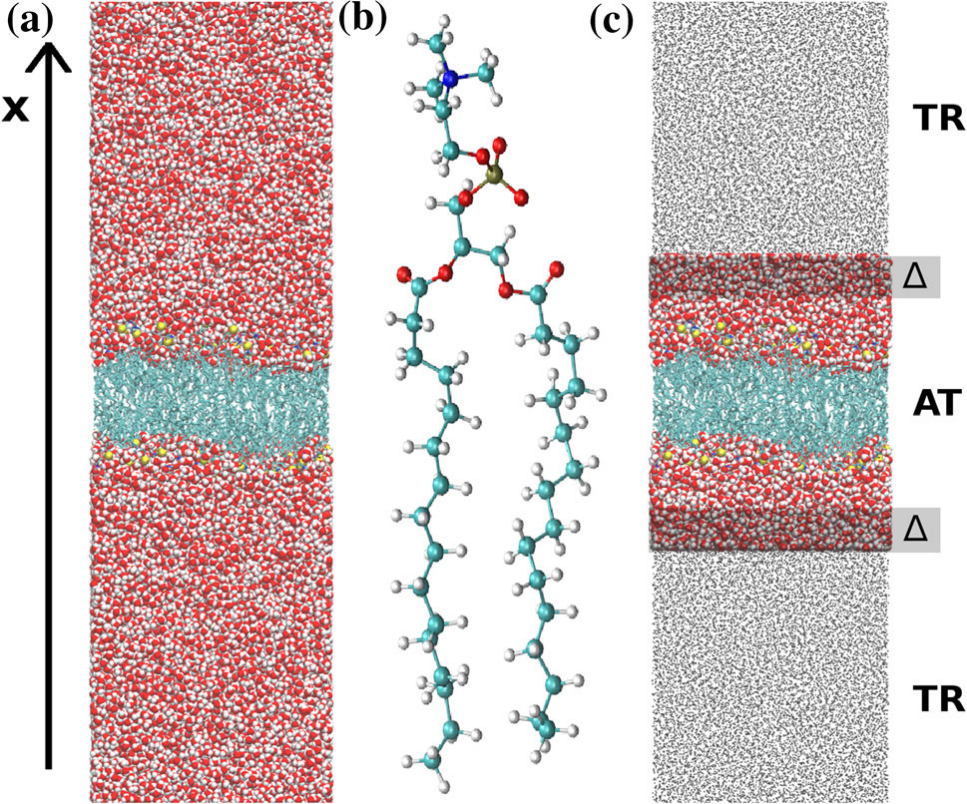
**a** Atomistic system of reference. **b** Atomistic structure of the DPPC molecule. **c** AdResS system with the DPPC bilayer oriented in the *z*–*y* plane and hydrated on top and bottom (along the *x*-axis). This figure is reproduced from Ref. [77]

In Ref. [77] the AdResS approach with tracers has been applied to the hydration of a DPPC bilayer. The atomistic system of reference consists of a bilayer of 180 (90 per leaflet) DPPC molecules, solvated with 52470 water molecules (see Fig. 12). By systematically reducing the AT region the authors in a quantitative manner, determined the extension of the region of this fluctuating system, where atomistic degrees of freedom are mandatory. Beyond that region a generic thermodynamic bath without atomistic details is sufficient. This analysis directly addresses the role of hydrogen bonding of the solvent on the stability of the membrane beyond a certain distance from the membrane-water interface. As a result this provides a definition of the size of the effective hydration shell of the membrane. Usually it is assumed that the first minimum of the radial distribution function of water as a function of the distance from the membrane is an optimal criterion to define the hydration layer [78]. In contrast, several recent studies have shown that water properties are influenced by biomolecules for distances beyond those defined by such a criterion [79]. The AdResS analysis, by identifying the minimal AT region, of about $0.7 \text {nm}$ from the surface of the membrane, within which the membrane has a direct effect on the structural properties of water (and vice versa), reproduces results of a reference full atomistic simulation and of recent experiments (see the detailed discussion in Ref. [77]).

#### Continuum hydrodynamics and open boundary molecular dynamics (OBMD)

**Fig. 13:**
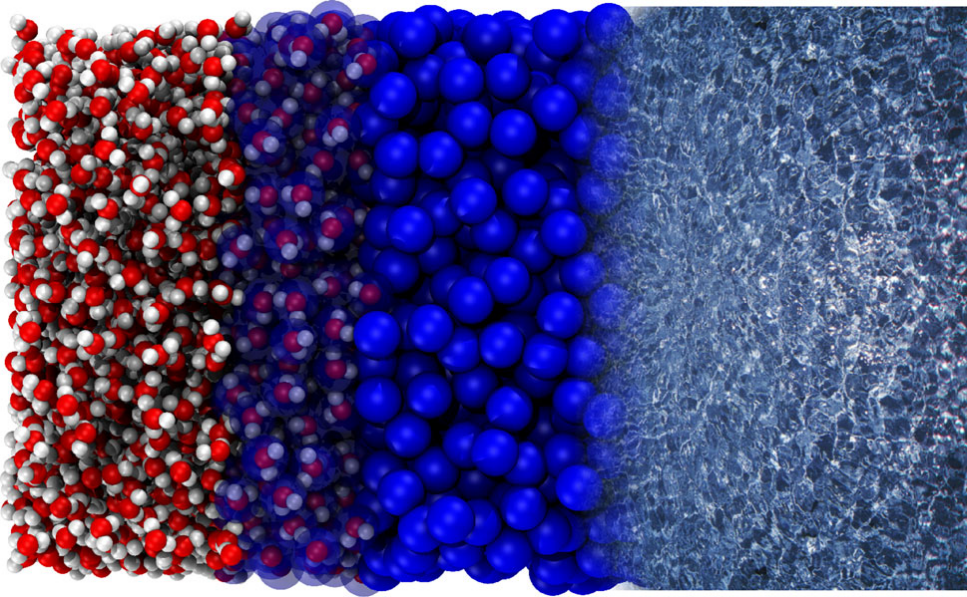
A triple-scale model of liquid water, which couples atomistic and continuum hydrodynamics. The particle region is simulated by MD and contains atomistic and coarse-grained water molecules. The water dynamics in the continuum domain is on the other hand governed by computational fluid dynamics equations. Figure reproduced from Ref. [91]

The AdResS concept as open boundary MD (OBMD) can be used to couple MD with computational fluid dynamics methods [80–88] resulting in a triple-scale setup [89, 90] as shown in Fig. 13 for liquid water. The purpose of the triple-scale scheme is twofold. It allows the coupling of a smaller AT region to a hydrodynamic continuum and at the same time also offers a way for insertion of complex molecules into a dense liquid, which would otherwise be difficult. The molecules can be easily inserted into the coarse-grained domain owing to soft effective interactions. As they move toward the atomistic region they acquire the missing fine-grained degrees of freedom courtesy of AdResS. The triple-scheme can the be used to simulate fluids under general external conditions, either in equilibrium or out of equilibrium, e.g., for simulation of the Couette and Stokes flows [89, 90]. Hybrid MD/CFD methodologies of this kind are relevant for instance for simulations of nano-technological or biomedical problems [92].

**Fig. 14:**
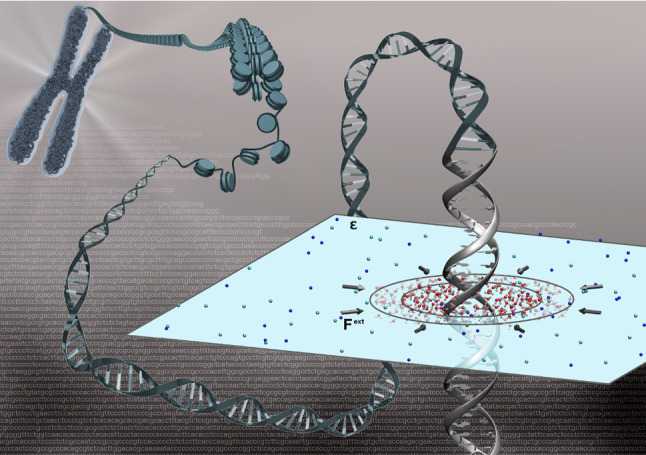
Cartoon representation of an atomistic DNA molecule immersed in the hybrid explicit/implicit salt solution simulated by OBMD. Figure reproduced with permission from Ref. [96], Copyright 2018, Biophysical Society

The above OBMD ansatz to include hydrodynamic interactions has been extended systematically [91, 93–95] beyond the grand-canonical ensemble towards non-equilibrium fluid flow simulations. Flow is introduced as an external boundary condition while the equations of motion for the bulk remain unaltered. To demonstrate its applicability, an example from Ref. [96], where OBMD was applied to a DNA molecule embedded in a hybrid explicit/implicit salt solution as shown in Fig. 14 is discussed.

The DNA molecule is modeled at atomistic resolution with the center of the explicit region coinciding with the DNA’s center-of-mass (CoM). The solvent’s level of representation depends on the distance from the DNA. At short distances, water is modeled with atomistic water model with ions explicitly present. Distal water is considered implicitly as a dielectric continuum, while the ions keep the original Lennard-Jones interaction parameters and charges as in the explicit region. However, the ion–ion electrostatic interactions are screened in the implicit region by the dielectric constant of water. In between the explicit and implicit water domains, there is a buffer region that acts as a reservoir. There, new water molecules are inserted, allowing the explicit domain to exchange mass, momentum, and energy through its boundary with the buffer. Water molecules that exit the buffer region into the implicit water domain are deleted.

The total force acting on a molecule $\alpha$ has three contributions

18 $$\begin{aligned} \mathbf{F}_{\alpha } = \mathbf{F}^{\text {AdResS}}_{\alpha } + \mathbf{F}^{\text {ext}}_{\alpha } + \mathbf{F}^{\text {DPD}}_{\alpha }. \end{aligned}$$

The adaptive resolution force $\mathbf{F}^{\text {AdResS}}$ accounts for the implicit/explicit resolution change, the external force $\mathbf{F}^{\text {ext}}$ imposes the desired external pressure tensor, and $\mathbf{F}^{\text {DPD}}$ is the force due to the linear-momentum-conserving dissipative particle dynamics (DPD) thermostat [97, 98].

The AdResS contribution on a molecule $\alpha$ is given by

19 $$\begin{aligned} \begin{array} {ll} \mathbf{F}^{\text {AdResS}}_{\alpha } &{} = \sum _{\beta \ne \alpha } \lambda (|\mathbf{R}_{\alpha }-\mathbf{R}|)\lambda (|\mathbf{R}_{\beta }-\mathbf{R}|)\mathbf{F}_{\alpha \beta }^{\text {ex}}\\ &{} + \sum _{\beta \ne \alpha } [1-\lambda (|\mathbf{R}_{\alpha }-\mathbf{R}|)\lambda (|\mathbf{R}_{\beta }-\mathbf{R}|)]\mathbf{F}_{\alpha \beta }^{\text {im}} \\ &{} - \mathbf{F}_{\alpha }^{th}(|\mathbf{R}_{\alpha }-\mathbf{R}|)\\ \end{array}\nonumber \\ \end{aligned}$$

where $\mathbf{F}_{\alpha \beta }^{\text {ex}}$ and $\mathbf{F}_{\alpha \beta }^{\text {im}}$ are the forces between molecules $\alpha$ and $\beta$, obtained from the explicit all-atom and implicit potentials, respectively. The $\mathbf{F}_{\alpha \beta }^{\text {ex}}= \sum _{i \in \alpha , j \in \beta } \mathbf{F}_{i\alpha j\beta }^{\text {ex}}= - \nabla _{\mathbf{r}_{ij}} U^{\text {ex}} (\mathbf{r}_{ij})$, where the sum runs over of all pair interactions between atom *i* of the molecule $\alpha$ and atom *j* of the molecule $\beta$. Note that the $\mathbf{F}_{\alpha \beta }^{\text {im}} \ne 0$ only for the ion-ion interactions. Here, the hybrid domain overlaps with the buffer domain. $\mathbf{R}_{\alpha }$, $\mathbf{R}_{\beta }$ and $\mathbf{R}$ are two-dimensional (*x*, *y*) vectors of CoMs of molecules $\alpha$ and $\beta$, and the DNA molecule, respectively. The thermodynamic (th) force $\mathbf{F}_{\alpha }^{\text {th}}$ acts on molecules’ CoM in the hybrid region and enforces a uniform density profile by compensating the chemical potential differences between the implicit and explicit resolution molecular models. The thermodynamic force depends on the molecule type, i.e., two different ones that correspond to Sodium and Chloride ions are used whereas for water, $\mathbf{F}_{\text {water}}^{\text {th}}=0$.

Water molecules are deleted once they leave the outer boundary of the buffer and new water molecules are inserted according to the desired average density in the buffer. The mass balance is controlled by a feedback algorithm, $\varDelta N_B = (\varDelta t / \tau _r)(\kappa (\langle N_B \rangle - N_B))$, where $\langle N_B\rangle$ and $N_B$ are the average and the current number of molecules in the buffer, $\kappa$ is a user-defined parameter, while $\tau _r$ is the characteristic relaxation time of the buffer. New water molecules are inserted if $\varDelta N_B > 0$. As they are inserted in the buffer that overlaps with the hybrid domain, the interactions with surrounding molecules are softer than in the explicit region.

The external boundary conditions are imposed on water molecules via $\mathbf{F}^{\text {ext}}$ computed from the momentum flux balance as

20 $$\begin{aligned} \mathbf{F}^{\text {ext}} = \mathbf{J} \cdot \mathbf{n}_B A + \frac{ \mathbf{P}_{\text {out}} - \mathbf{P}_{\text {in}} }{\varDelta t } + \sum _{\alpha }{} \mathbf{F}_{\alpha }^{\text {th}}, \end{aligned}$$

where $\mathbf{P}_{\text {out}}$ and $\mathbf{P}_{\text {in}}$ represent the total linear momenta of the water molecules that were removed and inserted into the simulation in the last time step of integration $\varDelta t$. $\mathbf{J}$ is the momentum flux tensor that one would like to impose across the boundary *B* of surface area *A*, $\mathbf{n}_B$ is the unit vector normal to the buffer interface. The last term is the total thermodynamic force with index $\alpha$ running over ions in the buffer domain. The total external force is distributed among the water molecules in the buffer $\mathbf{F}^{\text {ext}}=\sum _{\alpha \in B} \mathbf{F}^{\text {ext}}_{\alpha }$ with $\mathbf{F}^{\text {ext}}_{\alpha } = \frac{m_{\alpha }}{\sum _{\alpha \in B} m_{\alpha }} \mathbf{F}^{\text {ext}}$. The explicit region acts as an open system, which exchanges mass with its surroundings. Insofar the grand canonical OBMD described above differs from the AdResS approach coupling all-atom models to an ideal gas, presented previously. This allows for efficient molecular simulations of biomolecules solvated in salty solutions at variable ionic strength, e.g., physiological (0.15 M) ionic conditions. The significant computational speed-up is achieved due to the absence of explicit water molecules in the implicit region.

## AdResS beyond equilibrium

Analysing and steering systems out of equilibrium is of great relevance and a technique like AdResS can be of major help in simulating regions where the effect of imposed perturbations is more relevant. In the previous section, we have already discussed OBMD and its applications to non-equilibrium situations by coupling a particle based method to a continuum. In this section we now report various approaches and applications where the original (H-)AdResS setup itself can be employed to study non-equilibrium problems.

### Free energies and nonequilibrium work relations

The standard calculation of solvation free energies (SFEs), i.e. the difference in free energy between having a solute molecule in a solvent and in gas phase at a given temperature and pressure, requires a delicate modulation of physical interactions that heavily depends on the system under consideration [99–111]. In turn, once an AdResS setup has been prepared, SFEs of a solute can be identified with the work necessary to drag the solute across the simulation box where atomistic and ideal gas representations of the solvent coexist at constant temperature and chemical potential [26]. For an infinitely slow pulling speed, the applied work is equal to the SFE and the procedure is analogous to a thermodynamic integration calculation. For a finite pulling speed, the resulting work allows us to compute the SFE via nonequilibrium relations (Jarzynski equality [112–114] and Crooks fluctuation theorem [115]). This procedure has been used to compute SFEs that well agree with literature data available for water and urea molecules and, more importantly, anticipates the systematic investigation of arbitrarily large and complex molecules in solution [26].

### Poiseuille flow

The study of diverse non-equilibrium phenomena, including gradient-induced liquid flow and crystal nucleation, demands flexible and efficient computational approaches. Despite the significant effort devoted to developing such methods, we still face the same obstacle from the past: the simulation setup should consider the system in thermal and chemical equilibrium with an infinite reservoir of particles. Within the AdResS method, it is possible to guarantee this condition [63]. Furthermore, this approach is completely general, enabling the study of gradient-induced phenomena without using external driving forces and ensuring, on average, linear momentum conservation.

**Fig. 15:**
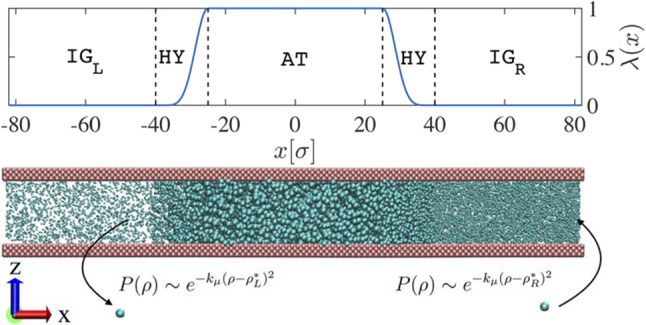
Adaptive resolution simulation setup used to investigate a LJ liquid confined between parallel plates under the effect of a density imbalance between right- and left-reservoirs. Reprinted from Ref. [63], with the permission of AIP Publishing

In practice, the simulation box contains a domain of interest, in which the description of the system is fully atomistic, and a reservoir of non-interacting, ideal gas, particles (Fig. 15). An external potential, applied only in the interfacial region, balances the excess chemical potential of the system. To ensure a density imbalance in the system, we use the particle insertion/deletion algorithm (Described in Sect. 4.2.2) to impose left $\rho _{\mathrm{{L}}}^*$ and right $\rho _{\mathrm{{R}}}^*$ reference densities.

This simulation setup allows one to sample non-equilibrium phenomena. In particular, we consider a prototypical LJ confined liquid under the influence of an external constant density gradient. The resulting pressure-driven flow across the atomistic system exhibits a velocity profile consistent with the corresponding solution of the Navier-Stokes equation [63]. The simplicity of the reservoir gives the possibility to study different out-of-equilibrium conditions for complex molecular systems, which constitutes a significant improvement over state-of-the-art simulation methods.

**Fig. 16:**
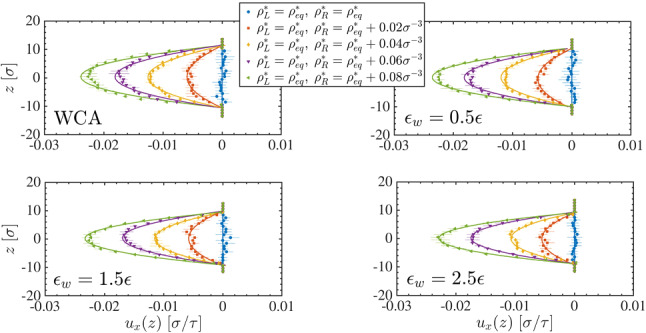
Velocity profiles of the pressure driven flow resulting from inducing different density gradients $\rho _{\mathrm{R}}^* \ge \rho _{\mathrm{L}}^* = \rho _{\mathrm{eq}}$ with $\rho _{\mathrm{eq}}$ the reference density at equilibrium. The solid lines are the parabolic fit to every data set. Reprinted from Ref. [63], with the permission of AIP Publishing

### Thermal gradient in open systems

**Fig. 17:**
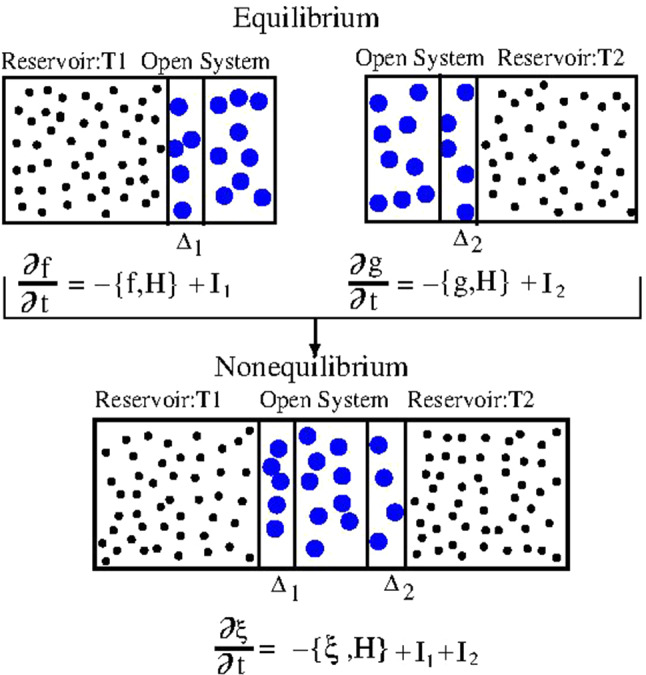
Pictorial illustration of the application of the linear action of the reservoirs in AdResS for the case of a thermal gradient. The system is first considered at equilibrium, at the thermodynamic condition of each reservoir, separately. Two separate simulations determine in AdResS $F^{1}_{\text {th}}(x)$ and $F^{2}_{\text {th}}(x)$. The open system is then put in contact with two different reservoirs; the mathematical models prescribe the linear action of the reservoirs: $I^{(1)}+I^{(2)}$. The linear action of the mathematical models in AdResS corresponds to the action of $F^{(1)}_{\text {th}}(x)$ and an external thermostat that keeps the temperature at $T_{1}$ in the region $\varDelta _{1}+\text {TR}_1$ and to the action of $F^{(2)}_{\text {th}}(x)$ and an external thermostat that keeps the temperature at $T_{2}$ in $\varDelta _{2}+\text {TR}_2$, that is: $I^{(1)}=F^{(1)}_{\text {th}}(x)+Thermostat\left( T_{1}\right)$ and $I^{(2)}=F^{(2)}_{\text {th}}(x)+Thermostat\left( T_{2}\right)$ so that $I^{(1)}+I^{(2)}=F^{(1)}_{\text {th}}(x)+Thermostat\left( T_{1}\right) +F^{(2)}_{\text {th}}(x)+Thermostat\left( T_{2}\right)$

**Fig. 18:**
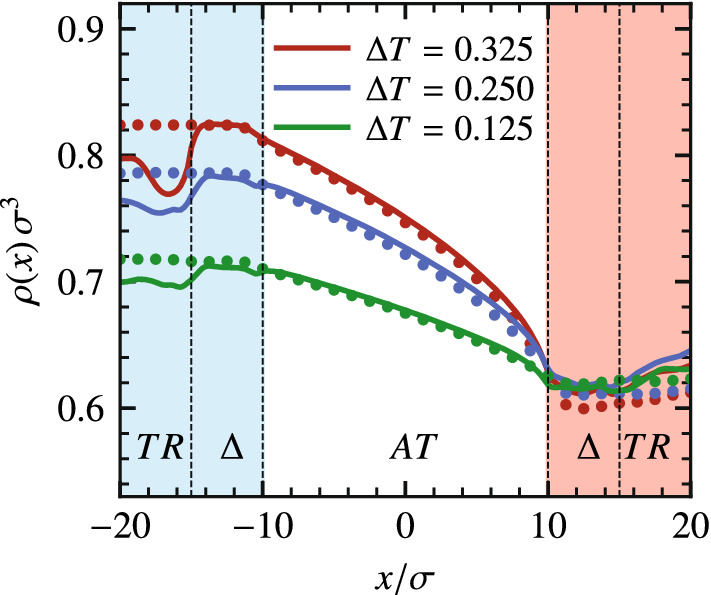
Density profiles for a liquid of Lennard-Jones particles in different thermal gradients, simulated with the setups of nonequilibrium AdResS (solid lines) compared with equivalent full atomistic simulations (dotted lines) in Lennard-Jones units. The gradient of temperature $\varDelta T$ between the hot (red) and cold (blue) reservoirs increases from bottom to top. In the atomistic region, i.e. the region of interest, the agreement with full atomistic results is highly satisfactory. This figure is taken from Ref. [36]

The previous examples, which are based on the application of the H-AdResS approach, demonstrate already opportunities AdResS offers to study non-equilibrium systems. More formally one can view e.g. Poiseuille flow simulations or similar situations as two combined AdResS simulations where the AT region couples to two different CG regions. This also can be formulated in terms of the Liouville operator theory shown in Sect. 4.1.2 as shown in Ref. [34] for embedding the AT system in different reservoirs [36, 72]. In fact both models imply, in first approximation, the linear sum of the actions of the different reservoirs in the Liouville equation for the distribution function of the open system. The action of each reservoir is expressed as if the system is in equilibrium at the system-reservoir coupling interface, e.g. two different reservoirs at different temperature, $T_{1}$ and $T_{2}$ (see also Fig. 17). First two separate AdResS simulations at equilibrium with the two different reservoirs are performed. From each of these simulations one obtains the respective different thermodynamic forces (or the free energy compensating functions). Next, the set up for the AdResS simulation out of equilibrium is prepared, that is the atomistic region is placed at the center of the simulation box and the two tracers regions (reservoirs), kept by a thermostat at temperature $T_{1}$ and $T_{2}$, are placed on the right and left side, respectively. The atomistic region is coupled to the reservoir at $T_{1}$ on the left side via a $\varDelta _{1}$ region where acts the thermodynamic force (or the free energy compensating function) obtained from the calculation at equilibrium with the reservoir at $T_{1}$; analogously for the other reservoir. This set up has been tested for Lennard-Jones fluids and it has shown to be very robust [72] even for particularly large temperature gradients [36] as illustrated in Fig. 18.

## AdResS at the quantum mechanical scale

So far the AdResS methodology has been discussed for classical systems, without considering any quantum degrees of freedom. Provided an appropriately adjusted coupling scheme can be constructed, there is no reason not to extend AdResS to the coupling between a classical and a quantum mechanical description. In this section some first results will be presented. The methodology can be rather straightforwardly extended to the path integral technique for the quantum description of light atoms and has been applied to liquid water and hydrophobic solvation. In parallel, ideas of including electronic degrees of freedom have been framed and are under development. Below we report the main ingredients of the two approaches and draw some future perspectives.

### Path integral AdResS

**Fig. 19:**
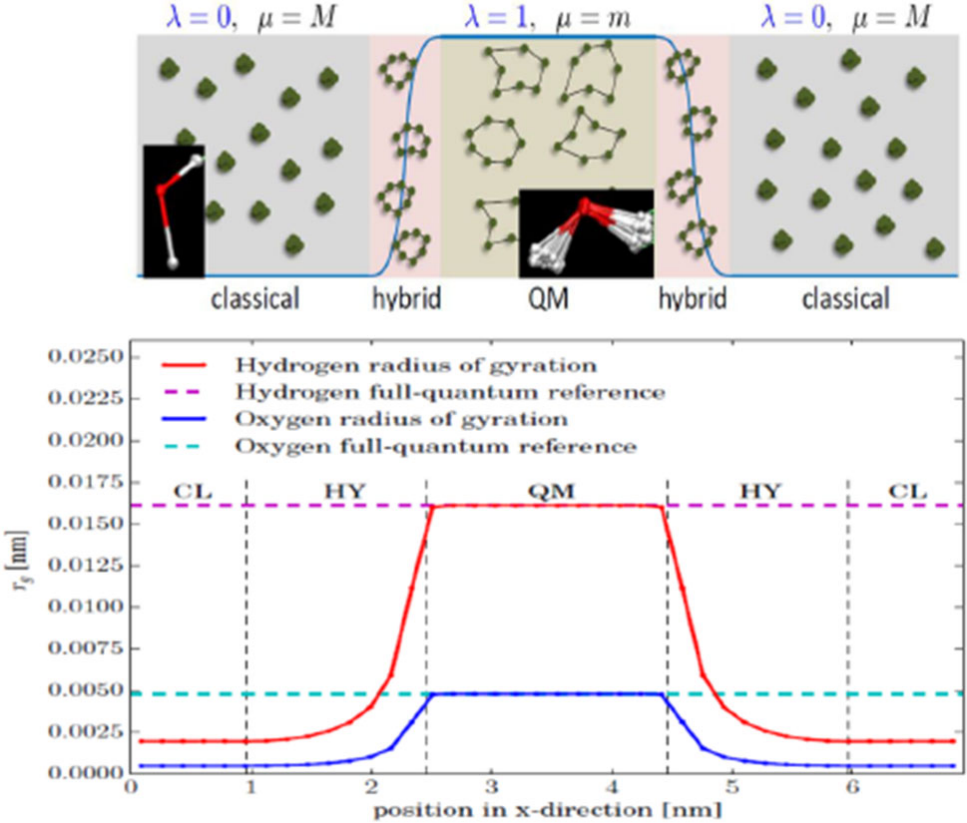
Pictorial illustration of AdResS with the path integral representation of the molecules in the high resolution region in the upper panel. The insertions show water molecules with the spatial delocalization of the hydrogens and the oxygens, respectively, in the different regions. The lower panel gives the radii of gyration of the polymer rings, representing the atoms, in the different regimes. The broken line corresponds to a full path integral quantum simulation of the whole system, demonstrating the excellent agreement. Figure adapted form [121, 122]

Path integral molecular dynamics (PIMD) is a well established technique taking advantage of the Feynman formalism [67]. Each atom, which in classical MD is a sphere, is delocalized over a polymer ring with *N* beads, each bead of the polymer is linked to its next neighbors along the chain by a harmonic potential. The interaction between atoms of different molecules takes place as in standard MD, via classical potentials, however the interaction is now distributed over all the beads of the quantum particle, typically the nucleus of a light atom. Moreover, differently from the standard ring–ring interaction, the ring–ring interaction in PI occurs only between corresponding beads of each of the interacting rings/atoms, e.g. between the bead *i* of atom *m* of molecule $\alpha$ and the bead *i* of atom *k* of molecule $\beta$: $r_{i,m,\alpha }$ and $r_{j,k,\beta }$ interacts if $i=j$. The deformation of the ring of each atom during the simulation mimics the quantum delocalization of the atom in space. At room temperature such an effect is only relevant for light atoms like the hydrogen. However, hydrogen bond networks in water-solvated systems and thus possible quantum effects of distortion of the network might be important in many areas of physics, chemistry and biology. Representing each quantum particle by *N* beads, and $N= 32, 64$ turn out to be reasonable choices, from the technical point of view requires at least *N* times the calculations of an equivalent classical system. Thus reducing the QM region, which is embedded in a classical environment, to a minimum would be a very appropriate application of the AdResS concept. As a consequence a PIMD AdResS could drastically reduce the cost of the simulation and deliver accurate results in the region of interest, e.g. around a solvated molecule. The algorithmic structure of AdResS, either with the thermodynamic force or with the free energy compensation, can be extended to the PI technique in a straightforward manner, since the polymer rings that represent the atoms are, from the simulation point of view, classical objects, and thus not different from AdResS simulation of classical molecules with multiple interaction sites [116–122]. The results for basic test systems and for liquid water at room temperature are excellent, as illustrated in Fig. 19. There for the quantum region the centroid MD has been used, where the delocalization of the nuclei is steered by a hypothetical position dependent mass $\mu$, while the mass for the advancement of the water molecules remains unchanged [121, 122]. For large $\mu$ the nuclei reduce almost to point particles, while for the physically correct mass in the QM regime the expected delocalization is observed. The extension to the solvation environment of a fullerene molecule in water demonstrates the general applicability. It was shown that the water structure around the fullerene in a classical simulations is more ordered and rigid than the structure found in quantum simulations [123]. The different rigidity of the solvating water might influence the actual aggregation barrier. The possibility of detecting such a quantum signature in the aggregation process is certainly a study where the AdResS technology shows its power and efficiency. In contrast to the path integral description, the inclusion of electronic degrees of freedom is not as straightforward, as shortly discussed next.

### AdResS for molecules with electrons

For AdResS with electrons a quantum region, treated with an electronic structure algorithm, is embedded in a classical environment treated via standard MD techniques. The forces acting on the atoms in the QM region come from the Hellmann-Feynman forces due to the electrons. The nuclei–nuclei interactions of the other atoms of the QM region and the interactions with the atoms composing the molecules of the classical environment can remain unaltered. Without any possible atom exchange between the quantum and the classical region such a setting is known as Quantum Mechanical/ Molecular Mechanics (QM/MM) method [124]. Recently Adaptive QM/MM (A-QM/MM) [125] has been introduced, where the process of exchange of molecules can be taken into account. The A-QM/MM methods mimics the variation of number of molecules in the QM region as described in the following. A buffer between the QM and the classical region is defined and at each time-step of the simulation, the molecules contained in the buffer are partitioned in all possible combinations of quantum and classical subsets, e.g. all molecules are labelled quantum, one is labelled quantum and the others classical, two are labelled quantum and the others classical....all molecules are labelled classical. For each partitioning the molecules in the subset labelled quantum are included in the QM region and treated quantum mechanically in the calculation. The total potential is defined as a weighted average of the individual potentials obtained in the simulations corresponding to each partitioning: $U(\mathbf{r})=\sum _{i}^{M}f_{i}(\mathbf{r})U_{i}(\mathbf{r})$, where $U_{i}(\mathbf{r})$ is the potential energy corresponds to one of the *M* partitioning of the system, $f_{i}(\mathbf{r})$ is a smoothing function depending on the coordinates of the single molecules. The smoothing function is based on the empirical idea that the quantum nature of molecules in the buffer at larger distance from the center of the region of interest is less relevant than the quantum nature of molecules closer to the active site. A first technical implementation of AdResS which follows this scheme has already been made with satisfactory results [126]. However A-QM/MM cannot be considered truly predictive because its results always require a case-by-case post-validation with larger QM calculations [125]. There are in fact several concerns, which question the applicability of this approach [127–129], most notably that interpolations of property-values between electron numbers may not consistent with a physical ensemble average [128, 129]. To overcome these drawbacks an el-Qm-AdResS with truly fluctuating electronic degrees of freedom is required. Such a theoretical model has recently been proposed [129, 130] and is currently under implementation. As in conventional AdResS and A-QM/MM of Ref. [126] thermodynamic equilibrium between the classical and the quantum region is mandatory, which leads to several specific requirements, namely

**Fig. 20:**
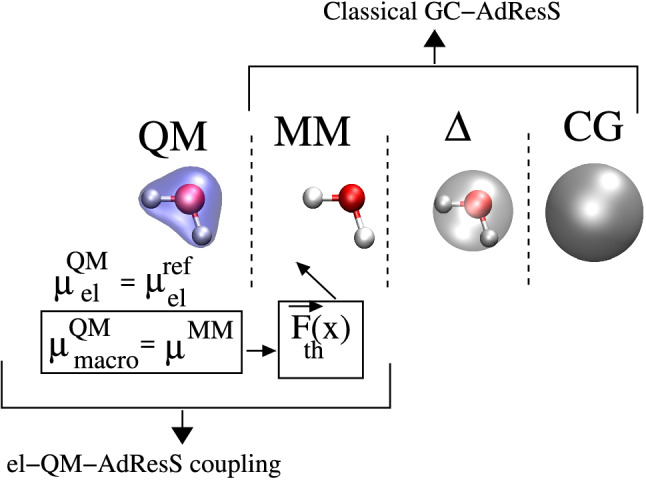
Pictorial representation of the el-QM-AdResS scheme. The macroscopic chemical potential ${\mu }_{\text {macro}}^{QM}$ is imposed by the thermodynamic force (or equivalently by the free energy balancing force) in the MM region and the $\varDelta$ region as in the classical AdResS scheme. ${\mu }_{\text {el}}^{\text {QM}}=\mu _{\text {el}}^{\text {ref}}$ indicates the electronic chemical potential of the QM region that must correspond to the chemical potential of the bulk in a full quantum calculation. Figure reproduced from Ref. [129], Copyright Wiley-VCH Verlag GmbH and Co. KGaA. Reproduced with permission

- The QM region is statistically well defined within a grand canonical ensemble [131] coupled to the classical environment.
- An appropriate thermodynamic force or free energy compensation is derived, as in standard AdResS and applied in the MM region at the interface with the QM region.
- electronic structure calculations are performed for the QM region via the grand-canonical energy functional minimization, at constant $\mu _{\text {el}}$, with the number of electrons being the variable [132, 133]

A pictorial illustration of the scheme is shown in Fig. 20.

## Conclusion/outlook

At the beginning of the new millennium the challenge of linking scales was identified as one of the highest priority for molecular simulation [134]. Though we are already 20 years into the first century this has not changed and demands are ever increasing. In phase with hard- and soft-ware development the need to correctly model ever more complex materials and systems is steadily rising. To meet the societal needs basically modified or new materials need to be developed. This demand certainly cannot be met without advanced computational methods, ranging from modern data driven methodologies all the way to quantum mechanical calculations. The development of AdResS follows from such a necessity and offered to the community an additional tool to tackle the scales interconnection that characterize complex molecular liquids and similar substances. The examples of applications reported here confirm the utility of the method in the context of molecular simulations. At the same time, besides the technical role of a useful algorithm, AdResS represents an ongoing theoretical challenge which requires to move out of conventional quantum and classical statistical mechanics and push towards more generic physical and mathematical models of systems that exchange energy and matter with the environment. In turn, the theoretical development inspires new numerical algorithms for the molecular simulation approaches that are becoming popular in the last years. In the era of machine learning and data driven models AdResS can certainly play a complementary role not only in the efficient production of data but also in their physical analysis. The detection of the locality of certain interactions or the identification of the relevance of some specific degrees of freedom over others can be achieved by applying AdResS and validated by the first principles on which the method is based. Interpreting the open systems aspect to calculate (solvation) free energies or using it to explicitly introduce non-equilibrium in a controlled manner points into new directions not anticipated in the very beginning. In this review we have attempted to give the overview of such concepts and to point to opportunities connected to adaptive resolution simulation methods like AdResS.

## Data Availability

This manuscript has no associated data or the data will not be deposited. [Authors’ comment: This is a review paper. The results shown belong to previously published work. For each of the previously published papers there is a proper corresponding statement about the data availability.]
